# Marine Food Polysaccharides: Representative Structural Features, Immunomodulatory Effects, and Food Applications

**DOI:** 10.3390/md24080286

**Published:** 2026-08-20

**Authors:** Yunhan Li, Xinhong Wang, Xiang Li, Nan Wei, Dawei Jiang, Ming Li, Chong Yu, Juan Guo, Keqiang Li, Haifeng Wang, Ning Li

**Affiliations:** 1 Department of Traditional Chinese Materia Medica, Shenyang Pharmaceutical University, Shenyang 110016, China; liyunhan594594@163.com (Y.L.); aaawxh0987@163.com (X.W.); 15042305472@163.com (X.L.); 15848255132@163.com (N.W.); yuchong0408@163.com (C.Y.); lkqphu@163.com (K.L.); 2 Zhangzidao Group Co., Ltd., Dalian 116200, China; 15094256198@163.com (D.J.); 15998408405@163.com (M.L.); 3 Dalian Institute of Marine Traditional Chinese Medicine, Dalian 116622, China; guojuanzy@163.com

**Keywords:** marine polysaccharide, structural features, immunomodulatory effects, functional applications

## Abstract

Marine food polysaccharides (MFPs) are structurally diverse biopolymers shaped by the marine environment, with emerging roles in immune regulation and food design. This review summarizes the representative structural features of MFPs, and evaluates their effects on innate and adaptive immune responses and associated signaling pathways. The influences of molecular weight, sulfation, branching, and monosaccharide composition on biological activity are also discussed. In addition, this review discusses the applications of MFPs in food additives, packaging, delivery systems, and functional foods. Despite growing evidence, challenges remain in resolving higher-order structures, understanding bidirectional immune effects and maintaining stability in complex matrices. Advancing integrated structural and food design approaches could enable the rational use of MFPs as next-generation functional ingredients.

## 1. Introduction

The ocean covers more than 70% of the Earth’s surface. It is the cradle of countless life forms and a vast reservoir of bioactive compounds. As the “blue granary,” the ocean provides abundant natural resources for the development of novel functional foods and drugs [1]. Blue Foods and the bioactive molecules they contain have emerged as an important frontier in modern nutrition and food science. Among these components, marine food polysaccharides (MFPs) have attracted considerable attention because of their distinctive chemical structures, diverse biological activities, and relatively low toxicity [2].

**Figure 1:**
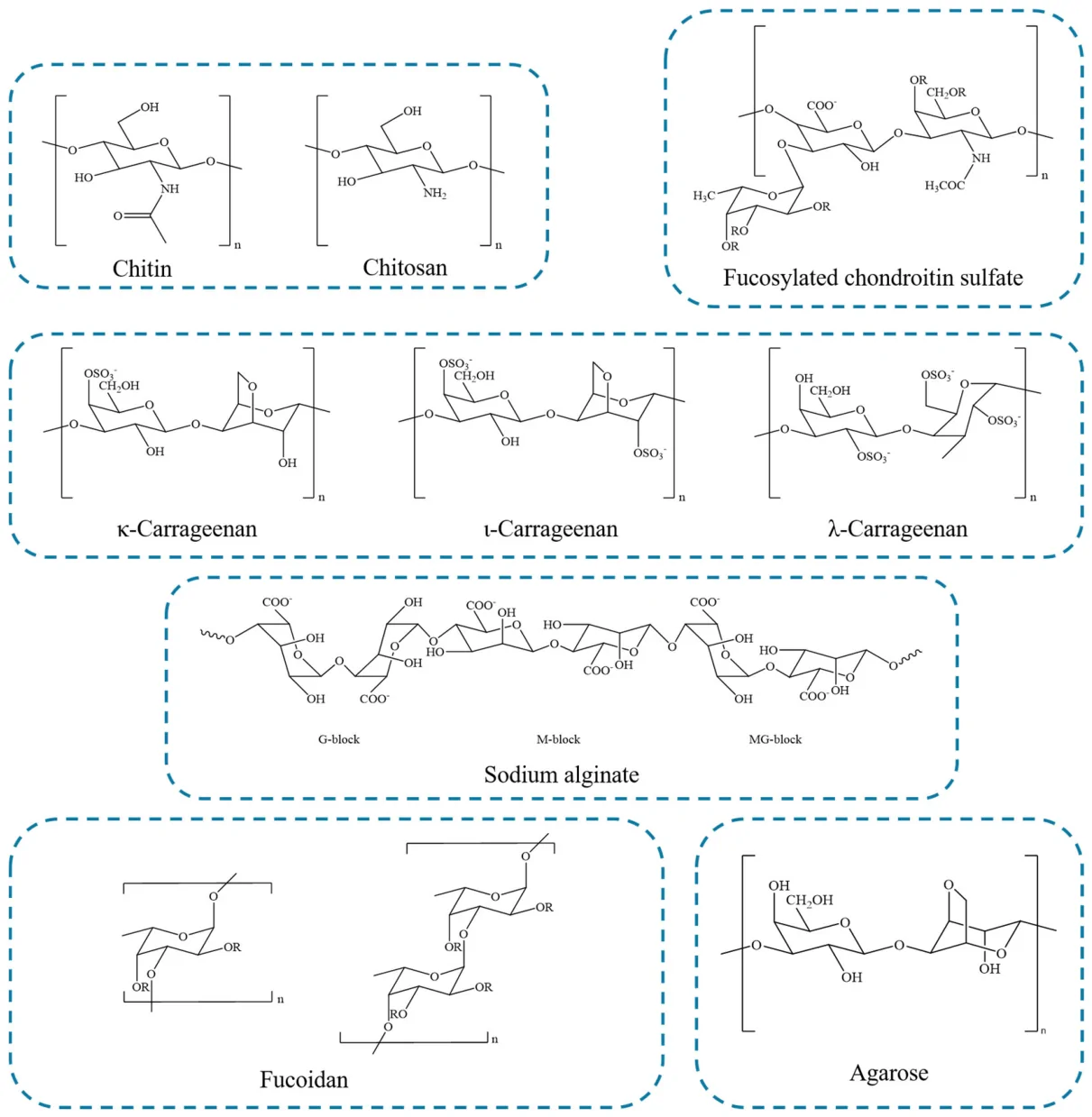
Structures of several typical MFPs. Note: R = H or SO_3_^−^.

China, as a major maritime country, possesses extremely rich marine biological resources. Among them, shellfish resources such as scallops, abalone, mussels, oysters, and clams have consistently ranked among the highest in global production for many years. These marine organisms contain a wide range of structurally novel polysaccharides that exhibit multiple biological activities, including anti-aging, antitumor, antiviral, immunomodulatory, hypoglycemic, and hypolipidemic effects [3]. In addition, crustaceans, fish, and marine algae also represent important sources of MFPs and provide key materials for the food industry, including alginate, carrageenan, agarose, chitosan, and glycosaminoglycans [4] (Figure 1). Compared with those land-derived polysaccharides, MFPs are influenced by high salinity, pressure, and low-temperature environments, which often make the structures of MFPs more complex and unique. Differences in monosaccharide composition, glycosidic linkage patterns, and sulfation modifications distinguish MFPs from the polysaccharides derived from terrestrial organisms [5]. These structural features not only confer unique physicochemical properties but also contribute to their significant potential in immunomodulation.

Increasing evidence indicates that food-derived bioactive compounds play an important role in maintaining immune homeostasis and preventing chronic inflammation-related diseases. Consequently, regulating immune responses through dietary bioactive components has gradually become an important focus in nutrition and health research [6,7]. MFPs can act as biological response modifiers and regulate immune responses through multiple pathways. They can be recognized by pattern recognition receptors located on the surface of immune cells, including Toll-like receptors such as TLR2 and TLR4 and the C-type lectin receptor Dectin-1, which subsequently activate intracellular signaling pathways such as NF-κB and MAPK. They can maintain internal homeostasis by regulating the functions of macrophages, T and B lymphocytes, and natural killer (NK) cells, as well as by activating the complement system. For example, sea cucumber polysaccharides have been shown to effectively promote the phagocytic activity of macrophages and stimulate them to release nitric oxide (NO), interleukin-6 (IL-6), and other cytokines [8].

Although many studies have reported the immunomodulatory activities and food applications of MFPs, it remains difficult to explain how differences in their structures contribute to the reported effects. This review mainly covers polysaccharides obtained from edible marine algae, including red, brown, and green algae, and marine animals. Marine-derived polysaccharides with clear relevance to food use are also involved. These include chitin and chitosan, marine glycosaminoglycans, particularly fucosylated chondroitin sulfate (FCS), carrageenan, alginate, fucoidan, agarose, and selected glucans and heteropolysaccharides. These polysaccharides were chosen because they are closely related to edible marine resources. Their structural features, immunomodulatory effects or food applications have been studied in reasonable detail (Table 1). The structural section mainly covers the relatively well-characterized groups, and other polysaccharides are included when they provide useful evidence on immunomodulation or food functions. This review summarizes their representative structural features, effects on innate and adaptive immunity, structure-activity relationships, and food applications, and discusses current limitations and future research needs. By critically examining and summarizing current research, and clarifying the relationships between structure and biological activity, this work aims to provide a solid theoretical basis for the further development and application of MFPs as functional food components and immunomodulatory agents.

The relevant literature for this review was collected from searches of the Web of Science, PubMed, and Scopus databases, with additional searches by using Google Scholar to identify potentially relevant studies that may have been missed in the major databases. The search terms mainly included “marine polysaccharides” or specific polysaccharide names (such as fucoidan, carrageenan, chitosan, glycosaminoglycans, and glucans), combined with keywords related to immunomodulation (such as immune regulation and immune cells) and food applications (such as food additives, functional foods, and food applications). The search covered publications available up to June 2026. Targeted supplementary searches, particularly for regulatory information, were conducted through July 2026. Recent peer-reviewed articles published in English were prioritized to reflect the current progress in this field. Meanwhile, some representative early studies that made important contributions to the structural characterization, biological functions, and food applications of MFPs were also included. The selection of references was mainly based on their scientific relevance, research quality, and contribution to this field. The reference lists of relevant articles were also examined to identify additional studies. When inconsistencies existed among studies, studies providing detailed structural characterization or mechanistic insights into biological activities were preferentially selected, and potential factors contributing to these differences were discussed in the manuscript.

## 2. Structural Features of Representative MFPs

Table 2 compares representative polysaccharide fractions whose molecular weight, monosaccharide composition, and glycosidic linkages have been characterized in detail. These examples are arranged according to their marine sources to show the structural diversity of polysaccharides obtained from algae and marine animals. As shown in Table 2, considerable structural variation exists not only among different groups of MFPs but also among polysaccharides obtained from similar marine sources. Algal polysaccharides frequently contain galactose, fucose, uronic acids, and sulfate groups, whereas animal-derived MFPs include glucans, amino-sugar-containing polysaccharides, and glycosaminoglycans. Their molecular weights and linkage patterns also vary widely.

### 2.1. MFPs Derived from Marine Animals

#### 2.1.1. Chitin and Chitosan

Chitin is an amino polysaccharide widely found in the shells of crustaceans such as shrimp and crab, as well as in the exoskeletons of mollusks such as abalone and squid. It is composed of N-acetyl-D-glucosamine (GlcNAc) units linked by β-(1→4) glycosidic bonds, in which the acetyl group substitutes the amino group at the C-2 position to form an amide bond (-NHCOCH_3_). In nature, chitin exists in α-, β-, and γ-forms. α-Chitin exhibits high crystallinity and mechanical strength due to its antiparallel chain arrangement. In contrast, β-chitin shows higher reactivity and hydration capacity owing to its looser structure [9].

Chitosan is a deacetylated derivative of chitin, consisting of randomly distributed GlcNAc and D-glucosamine (GlcN) units linked by β-(1→4) glycosidic bonds. Chitin with a degree of deacetylation (the percentage of β-1,4-D-GlcN units) above 50% is generally considered chitosan, and commercial chitosan typically has a degree of deacetylation ranging from 70% to 95%. The degree of deacetylation is an important structural parameter of chitosan. A higher degree of deacetylation usually results in higher solubility in acidic media and stronger biological activity [28]. This is because the higher degree of deacetylation increases the density of free amino groups along the chain; under acidic conditions these groups become protonated, which weakens interchain hydrogen bonding and promotes chain expansion in solution [29]. The resulting higher positive charge density also strengthens electrostatic binding to negatively charged microbial surface components such as lipopolysaccharides and teichoic acids, which is considered a key contributor to the enhanced antibacterial and immunomodulatory activities observed for highly deacetylated chitosan [30,31]. Near-neutral to basic pH favors a more compact, aggregation-prone arrangement via interchain hydrogen bonding [32]. However, the solubility and biological activities of chitosan are severely affected by pH, which limits its practical applications. To expand its functionality and application fields, researchers have carried out extensive chemical modifications on chitosan. Common modifications include carboxymethylation, quaternization, and alkylation, mainly targeting the amino group at the C-2 position of the deacetylated units and the hydroxyl groups at the C-3 and C-6 positions [33]. Carboxymethylation is typically achieved by reacting chitosan with monochloroacetic acid under alkaline conditions (NaOH). The reaction occurs mainly at the hydroxyl group of the C-6 position or simultaneously at the C-6 hydroxyl and C-2 amino groups, making chitosan with both anionic (-COO^−^) and cationic (-NH_3_^+^) properties. Carboxymethyl chitosan (CMC) exhibits significantly improved water solubility, chain flexibility, antibacterial, and antioxidant abilities [34]. Quaternization, typically achieved by reacting the C-2 amino group with glycidyltrimethylammonium chloride or methyl iodide, increases the cationic density, hydrophilicity, bioadhesiveness, and mucoadhesiveness of chitosan. This modification plays an important role in the development of biomedical materials such as wound dressings and drug delivery scaffolds. Alkylation, commonly achieved via reductive amination of the C-2 amino group with fatty aldehydes in the presence of sodium cyanoborohydride, improves the emulsifying ability and stability of chitosan, making it useful for developing emulsifiers [35]. In recent years, green approaches such as laccase- or tyrosinase-catalyzed enzymatic grafting and genipin-mediated cross-linking have gradually become research hotspots, providing new approaches for the sustainable utilization of chitosan in food and biomedical materials. However, chemical modification of chitosan still faces several challenges, including difficulty in precisely controlling the degree and site-selectivity of substitution, dependence on organic solvents or harsh reaction conditions that raise environmental and biocompatibility concerns, and batch-to-batch variability arising from the heterogeneous distribution of amino and hydroxyl groups along the chain [36]. Addressing these limitations through greener and more selective modification strategies remains an important direction for future research.

#### 2.1.2. Fucosylated Chondroitin Sulfate

Fucosylated chondroitin sulfate (FCS) is a unique glycosaminoglycan found in the body wall of sea cucumbers, with a molecular weight typically ranging from 20 to 100 kDa [37]. FCS possesses the same backbone as the conventional chondroitin sulfate (CS), which consists of repeating disaccharide units of β-D-N-acetylgalactosamine (GalNAc) and β-D-glucuronic acid (GlcA) in an approximate molar ratio of 1:1. GalNAc and GlcA are alternately linked by β-(1→3) and β-(1→4) glycosidic bonds, forming the backbone of FCS. Unlike the linear structure of CS, FCS also has a branched structure with fucose side chains. The fucosyl branching represents the defining structural distinction between FCS and CS. It is also regarded as the structural basis underlying the stronger and more diverse anticoagulant and immunomodulatory activities of FCS relative to conventional CS. The structures of these fucosyl branches exhibit considerable diversity. Typically, each side chain contains one fucose residue that is attached to the O-6 position of GalNAc or the O-3 position of GlcA through an α-(1→3)-glycosidic bond. In addition, some FCS molecules possess side chains composed of two or more linked fucose residues. More unusual side chain structures have also been reported [38]. In addition, some FCSs possess even more distinctive side chains. For example, FCSs isolated from *Psolus peronii* and *Holothuria nobilis* (PP and HN) contain a heterodisaccharide side chain of α-D-GalNAc4S6R-(1→2)-α-L-Fuc3S4R (R = H or SO_3_^−^) attached to the O-3 position of GlcA. These unusual side chains further expand the structural and functional diversities of FCS. PP and HN, which carry rare GalNAc–Fuc disaccharide branches, stimulated the proliferation of hematopoietic progenitor cells and lymphocytes more strongly than FCSs with only monofucosyl branches. This difference was associated with the abundance and sulfation pattern of the disaccharide branches, indicating that these features influence the biological activity of FCS. This may be because the longer disaccharide branches may present sulfate groups over a broader surface, favoring interactions with cell-surface proteins and contributing to the stronger hematopoietic response [39].

FCSs derived from different sea cucumber species and climatic regions exhibit variations in the degree and position of sulfation [40]. Sulfate groups are usually located at the C-4 and/or C-6 positions of the GalNAc residue and the C-2 position of the GlcA residue [41]. In some cases, substitution also occurs at the C-3 position of GlcA [42]. Fucose residues can also carry sulfate groups, forming mono- or disulfated structures at the C-2, C-3, and C-4 positions. Different sulfation patterns create distinct distributions of negative charge on the molecular surface, which can alter the affinity of FCS for protein receptors. FCS containing disulfated fucose branches generally exhibits a higher negative charge density and tends to interact more strongly with positively charged regions on receptor proteins, resulting in more pronounced biological activity than that observed for monosulfated forms. In addition to the degree of sulfation, the specific sulfation position also plays an important role in determining biological function. For instance, the sulfate groups in Fuc2S4S can form stable salt bridges with positively charged amino acid residues, such as arginine and lysine, on coagulation factor IXa. By contrast, Fuc3S4S has been reported to effectively inhibit pancreatic lipase and therefore exhibits potential activity in the regulation of lipid metabolism [43]. These observations suggest that different sulfation arrangements on the fucosyl side chains may induce local conformational variations in the polysaccharide chain, enabling the recognition of different protein targets and leading to diverse biological functions. Besides the sulfation of the side chains, the sulfation pattern of GalNAc residues in the main chain also affects the biological properties of FCS. FCS molecules containing a CS-E type backbone, characterized by GalNAc residues with 4,6-disulfation, exhibit a higher negative charge density. This highly sulfated structure may increase the extension of the polysaccharide chain and reduce steric hindrance between side chains, thereby facilitating the interaction between fucose residues and receptor molecules. In comparison, FCS containing CS-A type backbones with GalNAc 4-sulfation or CS-C type backbones with GalNAc 6-sulfation generally display lower charge densities and weaker electrostatic interactions with protein receptors [44]. These effects were directly compared in a series of enzymatically synthesized FCSs with comparable degrees of fucosylation but defined sulfation patterns on the fucose branches. Conversion of the branches from non-sulfated to 4-O-sulfated and then to 2,4-di-O-sulfated fucose progressively increased factor IXa affinity and anticoagulant activity. The 2,4-di-O-sulfated FCS interacted with positively charged Arg and Lys residues on factor IXa, showing how sulfate content and the additional 2-O-sulfate shape the protein-recognition pattern underlying the anticoagulant activity of FCS [45]. FCSs with a similar chondroitin sulfate backbone can display different solution conformations. fCS-Aj from *Apostichopus japonicus* adopted a random-coil conformation in PBS, whereas fCS-Hm from *Holothuria mexicana* formed a more extended linear chain and showed lower flexibility. The difference was associated with the combined effects of molecular weight, sulfate content, and sulfation pattern rather than with a single structural parameter [46]. Meanwhile, fCS-Sc from *Stichopus chloronotus* was characterized as a rigid rod-like chain with a single-helical structure [47]. These indicate that the higher-order structure of FCS should be interpreted in the context of its molecular size and the overall pattern of fucosylation and sulfation. Future studies are still needed to further elucidate the structural diversity of FCS and to perform systematic comparisons of FCS isolated from different sea cucumber species. Such efforts will help to establish a more comprehensive understanding of the structural characteristics of FCS and provide a stronger foundation for exploring its structure-activity relationships.

### 2.2. MFPs Derived from Marine Plants

#### 2.2.1. Carrageenan

Carrageenan is a class of sulfated linear polysaccharides widely found in the cell walls of red algae, with an average molecular weight ranging from 200 to 800 kDa [11]. It is mainly derived from red algae such as *Kappaphycus alvarezii* and *Gigartina* spp., serving as one of the primary structural components of these algae. The basic backbone of carrageenan consists of alternating β-D-galactose (Gal) and α-D-Gal units linked by β-(1→4) and α-(1→3) glycosidic bonds. Different numbers and positions of sulfate groups are substituted in the structure, and the degree of sulfation is about 15–40%. In some carrageenans, the hydroxyl groups at the C-3 and C-6 positions of α-D-Gal undergo dehydration to form an ether linkage, resulting in a 3,6-anhydro structure. Based on the differences in sulfation sites and the anhydro structure, carrageenan is classified into several types, among which κ-, ι-, and λ-carrageenans are the most commonly used in foods [48]. κ-Carrageenan carries one sulfate group at the C-4 position of β-D-Gal and contains a 3,6-anhydro structure in α-D-Gal, enabling the formation of firm gels. ι-Carrageenan has two sulfate groups located at the C-4 position of β-D-Gal and the C-2 position of α-D-Gal, and also possesses a 3,6-anhydro structure, forming more elastic and flexible gels. λ-Carrageenan contains three sulfate groups, at the C-2 position of β-D-Gal and the C-2 and C-6 positions of α-D-Gal, but lacks the 3,6-anhydro bridge, so it generally cannot form gels under conventional food-processing conditions [49]. At high temperatures, the chains of κ- and ι-carrageenan are mainly present in a disordered coil state. During cooling, the chains form double helices, which subsequently associate to form a gel network. The gelation of κ-carrageenan is promoted mainly by K^+^, whereas that of ι-carrageenan is more strongly affected by Ca^2+^ [50,51]. In contrast, λ-carrageenan has a higher sulfate content and lacks the 3,6-anhydrogalactose residue required for stable helix formation. It therefore remains mainly in a hydrated coil conformation [52]. The structural differences among these three types can be attributed to distinctions in their biosynthetic pathways. κ- and ι-Carrageenans undergo a process catalyzed by galactose sulfurylases that forms the 3,6-anhydro bridge, resulting in partial coiling of the molecular chains and gelation ability. In contrast, λ-carrageenan does not undergo dehydration; instead, it experiences high-sulfation at multiple positions catalyzed by carbohydrate sulfuryltransferases, which make its structure more flexible and hydrophilic but non-gelling [53]. Moreover, the structure of carrageenan is significantly influenced by algal species and environmental factors such as light, temperature, salinity, and harvesting season. These structural distinctions determine different applications of various types of carrageenan. κ-Carrageenan, which forms harder and stronger gels, is widely used as a stabilizer in dairy and meat products. ι-Carrageenan, which forms softer gels, can be used as a material for 3D food printing [54,55]. The more flexible λ-carrageenan can be used as a thickener and stabilizer in protein-containing food systems [56]. However, the potential negative effects of carrageenan on human health should not be ignored. Studies have shown that carrageenan can affect gut microbiota and may induce intestinal inflammation [57]. Therefore, future research should not only focus on exploring new applications of carrageenan in the food industry but also emphasize investigations into its structural safety, metabolic pathways, and health effects. Such studies will support the safer and more functional use of carrageenan in the food field.

#### 2.2.2. Sodium Alginate

Sodium alginate is one of the most important acidic polysaccharides in brown algae. It is mainly found in the cell walls and intercellular matrix of brown algae such as *Laminaria japonica* and *Sargassum* spp., and plays an important role in the development of food additives, wound dressings, and drug delivery systems. Sodium alginate is a linear copolymer composed of β-D-mannuronic acid (M units) and α-L-guluronic acid (G units) linked by (1→4) glycosidic bonds [58]. The monomeric residues occur as homopolymeric MM- and GG-blocks or alternating MG-blocks. The M/G ratio and block distribution strongly affect the physicochemical properties and functional performance of sodium alginate. The diaxial linkage between adjacent G residues gives GG-blocks a buckled conformation, whereas MM-blocks, with diequatorial linkages, adopt a flatter and more extended conformation. MG-blocks show intermediate structural characteristics [59]. These differences influence the affinity of the blocks for Ca^2+^. Accordingly, intrinsic chain rigidity generally follows the order GG > MM > MG [60]. G-rich alginates generally form stiffer and more thermally stable calcium gels, whereas M-rich alginates tend to form softer and more flexible networks. By selecting alginates with different M/G ratios and block distributions, gels with different mechanical strength, porosity, and release behaviors can be obtained for food coatings, food gels, and encapsulation systems. Each monosaccharide residue in sodium alginate contains a carboxyl group at the C-6 position, which can be protonated or ionized depending on the pH of the solution [61]. The carboxyl groups in the G-block can chelate with divalent cations, especially calcium ions (Ca^2+^). Ca^2+^ ions fit into the cavities between adjacent G units and coordinate with the carboxyl groups at their C-6 positions. Subsequently, neighboring egg-box units couple along the polymer chain to form a three-dimensional gel network based on the “egg-box model”, which can dynamically rearrange and self-heal. This structure endows sodium alginate with excellent viscoelastic properties and serves as the molecular basis for its application as a food gel and sustained-release carrier [62]. However, the presence of a large number of carboxyl and hydroxyl groups makes sodium alginate highly hydrophilic and easily soluble in water, which limits its application in aqueous environments. Hydrophobic modification of sodium alginate can overcome this limitation and expand its application range. Currently, reported chemical modification methods mainly focus on the carboxyl groups, including esterification, graft copolymerization, amidation, Ugi reaction, and stepwise covalent modification. There are also modification methods targeting hydroxyl groups, such as oxidation and graft copolymerization [12]. In addition, some physical and composite modification methods can also effectively regulate the hydrophobicity of sodium alginate, thereby broadening its application in various fields [63]. Although sodium alginate has a long history of application, several aspects still require further research. Future studies on its structure should be application-oriented, focusing on developing more effective approaches to adjust the M/G ratio and distribution to achieve precise molecular control. Meanwhile, most existing modification methods rely heavily on organic solvents, with several problems, such as environmental pollution, complex procedures and difficult purification, to be solved. Therefore, the development of greener and more efficient modification methods is needed. Promising greener alternatives currently being explored include enzyme-catalyzed esterification, solvent-free mechanochemical grafting, and ionic-liquid-mediated modification, which could reduce reliance on organic solvents while maintaining reaction efficiency and site selectivity. Moreover, the composite development and application of sodium alginate with other polysaccharides or proteins (such as chitosan, gelatin, and nanocellulose) may further broaden the range of obtainable material properties [64].

#### 2.2.3. Fucoidan

Fucoidan is a class of widely distributed sulfated heteropolysaccharides with complex structures that exhibit various effects on promoting health, including immunomodulatory, antitumor, antioxidant, and anticoagulant activities. It is abundantly present in the cell walls and intercellular matrix of brown algae, mainly derived from species such as *Laminaria japonica*, *Undaria pinnatifida*, and *Sargassum fusiforme*. The backbone of fucoidan is primarily composed of L-fucose residues, which are substituted to varying degrees by sulfate groups, and in some cases, acetyl groups. Unlike sulfated fucans from marine echinoderms, fucoidans from brown algae usually contain small amounts of other monosaccharide residues such as mannose, uronic acids, galactose, and xylose, bringing them more complex structures and more various activities. The backbone structure of fucoidan generally follows two connection patterns, one consisting of repeating (1→3)-α-L-Fuc linkages, and another with alternating (1→3)-α-L-Fuc and (1→4)-α-L-Fuc linkages. In addition, fucoidans often possess side chains of different lengths and substitution patterns, which may be composed of Fuc or other monosaccharide residues with various sulfate degrees, further influencing their structural diversity [13]. Monosaccharide composition is one of the key factors influencing the biological activity of fucoidan. Certain sulfated fucose patterns, such as fucose residues sulfated at the C-4 position, can mimic the binding patterns of endogenous ligands with their receptors. A higher fucose content is often associated with stronger anti-inflammatory and anticoagulant activities. At the same time, the presence of other monosaccharide residues can further modulate biological effects. Galactose residues can enhance the recognition of polysaccharides by specific lectin receptors, thereby promoting the activation of dendritic cells and natural killer cells. Fucoidans enriched in galactose, often referred to as galactofucoidans, show particularly strong antiviral and antitumor immunomodulatory activities [65]. Mannose residues may enhance immunoregulatory effects, while xylose residues have been associated with antioxidant activity. In addition, uronic acids introduce extra negative charges to the polysaccharide chain, which improves water solubility and strengthens electrostatic interactions with positively charged cytokines or chemokines [66].

The molecular weights of fucoidan vary widely from below 100 kDa to over 1500 kDa, depending on the source species, extraction and purification procedures, and analytical methods. These variations may contribute to differences in their immunomodulatory activities [16]. Studies have shown that molecular weight exerts a more significant impact on activity, compared with the degree of sulfation, branching pattern, or monosaccharide composition [67]. Due to its high viscosity and resistance to enzymatic degradation, high-molecular-weight fucoidan (HMWF) can often maintain an intact structure until it reaches the small intestine. After absorption, it can stimulate local macrophages and transmit signals to helper T cells, thereby modulating the intestinal immune microenvironment. In addition, the long polymer chains of HMWF allow simultaneous binding to multiple receptors on the surface of immune cells, such as TLR4. This multivalent interaction can promote receptor clustering and dimerization, which facilitates the activation of downstream signaling cascades [66]. In contrast, low-molecular-weight fucoidan (LMWF) exhibits better solubility, lower steric hindrance, and higher diffusion ability. Pharmacokinetic studies indicate that LMWF shows faster absorption, higher systemic exposure, and improved bioavailability compared with HMWF. As a result, LMWF can more easily cross capillary barriers and enter the bloodstream, after which it can be distributed to immune-related organs such as the liver and spleen as well as to other tissues [68].

Fucoidan is usually highly sulfated, with sulfate groups primarily located at the C-2 or/and C-4 positions of fucose residues, occasionally at the C-3 position, and in some cases showing 2,3- or 3,4-disubstitution. Generally, sulfation at the C-2 position is closely related to anticoagulant activity, whereas sulfation at the C-4 position is more strongly associated with anticancer activity [69,70]. The degree of sulfation is an important factor affecting the biological functions of fucoidan. Highly sulfated fucoidans generally display stronger anticoagulant, immunomodulatory, and antitumor activities, which can be attributed to their higher negative charge density that enhances interactions with positively charged biomolecules, such as cationic groups on cofactor II and Toll-like receptors [71]. However, there are also studies that have reported a negative correlation between sulfate content and the anti-lipogenesis activity of fucoidan in hepatocytes [72]. This may be because it requires fucoidan to enter hepatocytes to exhibit its anti-lipogenesis activity, while increased negative charge density reduces its membrane permeability and weakens its binding affinity to intracellular receptors. The specific mechanisms and pathways involved require further investigation. Moreover, the structural features of fucoidan also influence other biological activities. High fucose content and high sulfate content have been correlated with stronger antioxidant, antiviral, antifungal, and prebiotic activities, while molecular weight further modulates these effects depending on the specific target pathogen or pathway [73,74]. In a comparative analysis of six brown-algal fucoidans, including samples from *Laminaria digitata* and *Saccharina latissima*, most fractions existed as expanded flexible chains in PBS, and the conformation data were consistent with branched structures with partly long side chains [75]. By contrast, a highly branched fucoidan from *Laminaria japonica* adopted a compact sphere-like conformation in water [76]. These results suggest that branch architecture and molecular size can modify the degree of chain extension in aqueous solution. However, the independent contribution of sulfation remains difficult to determine because it commonly varies together with molecular weight and monosaccharide composition in natural fucoidans. Currently, research on structures of fucoidans mainly focuses on the primary structures, while studies on the higher structures remain limited. Future work should not only further summarize its primary structural features but also emphasize the analysis of its advanced structures. By integrating multidimensional spectroscopic techniques with molecular modeling, it will be possible to elucidate the conformational characteristics and dynamic behavior of fucoidan in solution and under physiological conditions. In addition, studies on structure-activity relationships should become a major research focus. Clarifying how molecular weight, sulfation sites and branching structures influence biological activity can provide a theoretical basis for the degradation, structural modification, and functional optimization of fucoidans, thereby further expanding its potential applications in food and health fields.

#### 2.2.4. Agarose and Agaropectin

Agar is a mixture of polysaccharides rather than a single polysaccharide. It is mainly extracted from red algae such as *Gelidium* and *Gracilaria* and consists chiefly of agarose and agaropectin. Agarose is the main component responsible for gel formation. It is a nearly neutral linear polysaccharide with agarobiose as its repeating unit. Each agarobiose unit contains β-D-galactose and 3,6-anhydro-α-L-galactose, and its backbone can be expressed as [→3)-β-D-Gal*p*-(1→4)-3,6-anhydro-α-L-Gal*p*-(1→]_n_ [77]. In hot water, the agarose chains become disordered. When the solution cools, the chains associate into helices and gradually form a three-dimensional network through hydrogen bonding, giving agar its thermoreversible gel properties. Agaropectin has a galactose-based backbone similar to that of agarose but contains more sulfate, pyruvate, and methyl substituents. Its structure is less regular and varies among algal species. The charged groups weaken the association between polysaccharide chains, so agaropectin contributes less to gel formation [78].

The gel strength and gelling temperature of agar are influenced by the relative amounts of agarose and agaropectin. Molecular weight, 3,6-anhydrogalactose content, sulfate content, and substitution patterns can also influence the resulting gel. In general, a higher content of 3,6-anhydrogalactose and a lower degree of sulfation favor more regular chain association and stronger gel formation. Alkali treatment is commonly used to improve the gel properties of agar. For agar obtained from *Gracilaria*, alkali treatment can convert part of the L-galactose-6-sulfate into 3,6-anhydro-L-galactose, thereby reducing the sulfate content and improving gel strength [79].

## 3. Immunomodulatory Effects of MFPs

The structural diversity of MFPs provides an important basis for their different immunomodulatory effects. Variations in molecular weight, sulfation, branching, and monosaccharide composition can influence their interactions with immune cells and the subsequent cellular responses. This section summarizes the effects of MFPs on innate and adaptive immunity and discusses major signaling pathways involved. Table S1 summarizes representative studies according to the main immune targets, including macrophages, NK cells, the complement system, and T and B lymphocytes. The experimental models, doses, biological effects, signaling pathways, and available toxicity information are compared to show both the common findings and differences among studies.

### 3.1. Regulation of Innate Immunity

**Figure 2:**
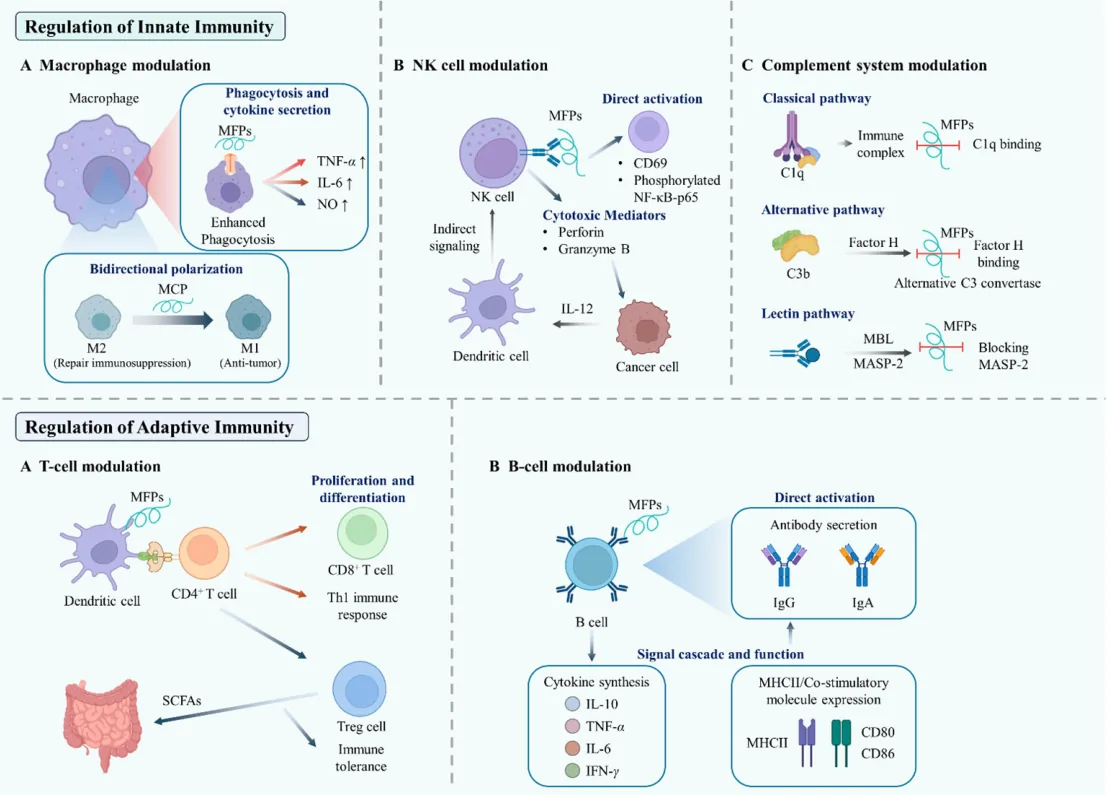
The immunomodulatory effects of MFPs in innate and adaptive immunity.

MFPs primarily regulate innate immunity by influencing macrophages, NK cells, and the complement system (Figure 2).

#### 3.1.1. Macrophage Modulation

Macrophages are key cells in the innate immune system. They play crucial roles in pathogen phagocytosis, antigen presentation, and cytokine secretion. MFPs have been widely reported to regulate macrophage functions through multiple pathways, thereby enhancing innate immune responses or maintaining immune balance.

Phagocytosis is one of the fundamental functions of macrophages, reflecting their capacity to recognize and eliminate foreign pathogens. Immunomodulatory effects of MFPs on macrophages show two main response patterns. In immunosuppressed or unstimulated models, MFPs generally restore macrophage activity. PHP from *Porphyra haitanensis* significantly enhanced the phagocytic activity of macrophages from hydrocortisone-induced immunosuppressed mice. High doses of PHP also increased lactate dehydrogenase (LDH) and acid phosphatase (ACP) levels, indicating a marked elevation in macrophage activation levels [80]. MFPs can significantly enhance the production of NO, iNOS, TNF-α and IL-6, reflecting their capacity to stimulate immune cell activation. Similarly, a series of glucans, HPP-1S to HPP-8S from *Hemicentrotus pulcherrimus* significantly increased TNF-α and IL-6 secretion and promoted NO release [81]. These α-glucans had similar backbones and were evaluated under the same conditions. HPP-6S had the highest degree of branching and showed the strongest activity. This may be attributed to its higher degree of branching, which increases contact with receptors. However, it still needs to be further examined separately.

In inflammatory models, the response is different. A highly branched polysaccharide, LJPS, from *Laminaria japonica*, showed a bidirectional regulatory effect on NO secretion. Under normal conditions, LJPS induced NO production in RAW 264.7 macrophages. However, under lipopolysaccharide (LPS) stimulation, LJPS suppressed excessive NO production [82]. This bidirectional effect may be related to the highly branched structure of LJPS, which may lead it to interact distinctly with macrophages under different immune microenvironments. It may therefore differentially regulate NO production and contribute to the maintenance of immune homeostasis.

Macrophages can polarize into two phenotypes in response to different microenvironmental signals: the M1 phenotype, which is pro-inflammatory and anti-tumor, and the M2 phenotype, which promotes tissue repair and tumor immunosuppression. An acidic heteropolysaccharide, MCP, extracted from *Ruditapes philippinarum* showed another form of immunomodulation. MCP not only induced differentiation of naive macrophages (M0) into M1-type macrophages but also reprogrammed M2-type macrophages into the M1-type macrophages [83]. Conversely, LJP61A, a homogeneous polysaccharide from *Laminaria japonica*, decreased the expression of M1 macrophage markers and increased that of M2 markers in Ox-LDL-stimulated macrophages [84]. These suggest that MFPs can modulate macrophage polarization in a context-dependent manner.

Overall, the regulation of macrophages by MFPs is better interpreted as an adjustment toward a response suited to the immune state, rather than as general stimulation or inhibition. This may help guide the development of functional foods based on MFPs for different immune needs.

#### 3.1.2. Natural Killer (NK) Cell Modulation

Natural killer (NK) cells, which eliminate virus-infected and tumor cells through direct cytotoxicity and cytokine secretion, are a vital component of the innate immune system. MFPs can significantly enhance NK cell immune functions through either direct activation or indirect signaling modulation.

MFPs can directly modulate NK-cell activity through interactions with cell-surface receptors and can significantly enhance their cytotoxic functions. Fucoidan (FDA) from *Durvillaea antarctica* increased CD69 expression and NF-κB p65 phosphorylation in NK cells, while also stimulating type 2 dendritic (cDC2) cells to produce IL-12 [85]. The release of cytotoxic mediators such as perforin and granzymes is one of the key mechanisms through which NK cells eliminate target cells. Sulfated polysaccharide CFP from *Codium fragile* significantly increased the production of perforin and granzyme B in splenic NK cells, thereby exhibiting stronger cytotoxicity against CT-26 tumor cells [86]. MFPs can indirectly enhance NK cell activity by improving the secretion of certain cytokines. A glucan (SEP) derived from *Strongylocentrotus nudus* eggs can increase the secretion of interleukin-2 (IL-2) and interferon-γ (IFN-γ), indirectly enhancing the cytolytic response of NK cells [87]. Some MFPs also affect the expression of activating and death ligands on the NK cell surface. Laminarin enhanced the expression of the activating receptors NKp30 and NKG2D in NK92-MI cells and increased NK-cell cytotoxicity, suggesting that it may improve the recognition of target cells by NK cells [88]. Fucoidans from *Fucus vesiculosus*, *Undaria pinnatifida*, *Ascophyllum nodosum*, *Macrocystis pyrifera*, and *Ecklonia cava* all upregulated the activating receptor NKp30, and the death ligands FasL and TRAIL on NK cells [89].

In a randomized pilot study, fucoidan from *Cladosiphon okamuranus* increased NK-cell activity after eight weeks, but a significant difference from the placebo group was observed only in male participants [90]. Therefore, the effects of MFPs regulating NK cells observed in cell and animal models are relatively consistent, but the effect in humans still requires confirmation in larger populations.

#### 3.1.3. Complement System Modulation

The complement system consists of soluble and membrane-bound proteins, which can be activated through the classical, alternative, and lectin pathways to clear pathogens by lysis and opsonization. The activation of the complement system must be tightly controlled, since excessive activity damages host tissue and drives inflammatory disease. Since most MFPs carry negative charges, they often exhibit potent anticomplementary effects by mimicking the polyanionic structures on host cell surfaces, thereby interfering with complement component binding or the regulatory activity of Factor H. In addition, some MFPs can also enhance complement activation, demonstrating bidirectional regulatory potential.

The classical complement pathway is usually initiated by the binding of C1q to immune complexes. This pathway serves as a critical bridge between innate and adaptive immunity. Sulfated fucans and sulfated galactofucans from *Sargassum thunbergii* can effectively inhibit the hemolytic reaction mediated by the classical pathway [91]. The inhibitory activity of MFPs against the classical pathway is associated with their structural characteristics, such as molecular weight, sulfate content, and uronic acid content. Studies have shown that anticomplementary activity increases with higher molecular weight and sulfate content [92]. This may be because the binding of C1q to antibodies in the classical pathway relies on the recognition of negatively charged regions by cationic amino acid residues. Polysaccharides with higher molecular weight and sulfate content possess larger and more negatively charged domains, which can effectively mask antibody-binding sites and block the formation of the C1 complex. Meanwhile, since uronic acids themselves do not exhibit anticomplementary activity, polysaccharides with higher uronic acid content show relatively lower activity due to the reduced proportion of other active components.

The alternative pathway is activated independently of antibodies and amplifies complement responses through continuous C3 activation. Crude polysaccharides (KCA and KCW) from *Kjellmaniella crassifolia* show much stronger inhibition of hemolysis than heparin. Sulfate groups are essential for maintaining this activity, as desulfated derivatives (ds-DKCA and ds-DKCW) show markedly reduced or even abolished activity [93].

The lectin complement pathway is triggered by mannose-binding lectin (MBL), which recognizes glycosylation patterns on pathogen surfaces and initiates a cascade reaction. MFPs can suppress this pathway by interfering with the recognition mediated by MBL. Carrageenans derived from red algae can inhibit the binding of C4 to mannose-coated surfaces. Among them, highly sulfated, non-gelling λ/μ/ν-carrageenans exhibit the most significant inhibitory activity, suggesting that a higher degree of sulfation facilitates interactions with MBL-related components and suppresses lectin pathway activation [94]. This may be because highly sulfated λ/μ/ν-carrageenans mimic the charge and spatial configuration of pathogen carbohydrate chains, which gives them the ability to compete with pathogen-associated carbohydrates for MBL binding, thereby preventing downstream complement activation. However, because the study measured downstream C4 deposition rather than direct binding to MBL, competitive recognition by MBL remains a possible mechanism which needs to be further studied.

Although most MFPs exhibit pronounced anticomplementary activity, some are capable of activating complement responses and promoting immune defense. Polysaccharides (ULP) from *Ulva lactuca* can induce macrophages to express the immune molecule CD5L, which in turn activates the complement cascade and significantly upregulates the expression of key membrane attack complex (MAC) proteins such as C5b-9, C6, and C9 [95]. Notably, these apparently opposite effects on the complement system of MFPs are not necessarily contradictory. Studies based on hemolysis mainly evaluated the ability of MFPs to reduce complement-mediated lysis. The study of ULP examined complement activation associated with tumor-cell killing. Therefore, the functional value of complement modulation by MFPs depends on the specific context. Inhibition may help limit excessive complement-mediated damage, whereas activation may strengthen complement-dependent immune defense.

### 3.2. Regulation of Adaptive Immunity

#### 3.2.1. T-Cell Modulation

T cells are a crucial type of lymphocyte in adaptive immunity. They play vital roles in maintaining immune balance, defending against pathogens, and performing immune surveillance, forming the core of cell-mediated immune responses. MFPs can regulate activation, proliferation, differentiation, and cytokine secretion of T cells, thereby influencing the direction of adaptive immune responses and maintaining immune balance.

MFPs can indirectly promote functions of T cells by enhancing the functions of antigen-presenting cells, such as dendritic cells. Fucoidan from *Undaria pinnatifida* promotes dendritic cell maturation and enhances the proliferation and activation of CD4^+^ and CD8^+^ T cells, thereby inducing a Th1-type immune response [96]. Similarly, Fucoidan FDA from *Durvillaea antarctica* induced TLR4-dependent dendritic-cell activation and increased IFN-γ-producing CD4^+^ and CD8^+^ T cells in mice [97]. These findings support that dendritic-cell activation is an important route through which fucoidans regulate T-cell responses.

Tregs are essential for controlling the intensity of immune responses, maintaining immune tolerance. MFPs can induce Treg cell differentiation to exert immunoregulatory effects and maintain immune homeostasis. A chondroitin sulfate (SCCS) from sea cucumbers increased IL-10 and Foxp3^+^ Treg cells and also altered the gut microbiota and SCFA production. This helps suppress excessive inflammation and establish immune tolerance [98]. Fermented *Gracilaria lemaneiformis* polysaccharides similarly inhibited Th2 differentiation and IL-4 production while promoting Treg differentiation and IL-10 production. The fermented preparation was more effective than the unfermented polysaccharide from the same source, suggesting that fermentation may provide a practical means of controlling the T-cell-modulating activity of MFPs during the development of functional foods [99].

#### 3.2.2. B-Cell Modulation

As a key component of the adaptive immune system, B lymphocytes (B cells) are primarily responsible for secreting antibodies and regulating immune response. MFPs can modulate the activation, proliferation, and antibody secretion of B cells, thereby regulating the different stages of humoral immune function.

MFPs can directly or indirectly stimulate the proliferation of B cells. The sulfated polysaccharide UCP from the green alga *Ulva conglobata* Kjellman and fucoidan DAP4 from the brown alga *Durvillaea antarctica* significantly stimulate lipopolysaccharide (LPS)-induced proliferation of B cells [100,101].

The activation of B cells by MFPs is often accompanied by increased expression of specific surface markers and elevated production of immunoglobulins (Igs). The sulfated polysaccharides FCSPs from the brown alga *Saccharina latissima* can upregulate the expression of the early activation marker CD69 on B cells [102]. Polysaccharide CLP-80 from *Caulerpa lentillifera* can upregulate the expression of co-stimulatory molecules CD80 and CD86. CLP-80 can also significantly increase serum levels of IgA and IgG in mice [103]. However, in a murine food-allergy model, *Ulva*-derived polysaccharide decreased serum IgG1 and OVA-specific IgE while increasing OVA-specific IgG. Therefore, a beneficial humoral response caused by MFPs may involve changing the antibody profile rather than simply increasing total antibody production [104].

In addition, MFPs do not always promote the proliferation of B cells and increase antibody responses simultaneously. Fucoidans from *Cladosiphon* and *Fucus* stimulated B-cell proliferation but inhibited IL-4- and CD40-induced IgE production by suppressing Cε germline transcription and NF-κB p52 nuclear translocation [105]. This also shows that the effects of MFPs on humoral immunity are more selective than a general increase in immune activity. However, the selective regulation has so far been demonstrated for only a limited number of MFPs and requires further investigation.

### 3.3. Signaling Pathways and Putative Mechanisms

The charged groups, such as sulfate and carboxyl groups of MFPs, can interact with pattern recognition receptors through electrostatic attraction. As a result, MFPs can activate key signaling pathways, such as NF-κB and MAPK, and trigger downstream kinase cascades. These pathways are involved in the immunomodulatory effects of MFPs.

#### 3.3.1. Receptor Recognition

The charged groups of MFPs can facilitate their initial contact with immune receptors, but receptor recognition also depends on the organization of the polysaccharide chain. In addition to the charge carried by sulfate and carboxyl groups, the glycosidic backbone, branching pattern, molecular size, and chain conformation affect how MFPs are presented to the receptor surface.

Dectin-1 exhibits stringent stereospecific requirements for glucan recognition. The interaction between β-glucans and Dectin-1 has been examined in detail. Laminarin induced cooperative tetramerization of the Dectin-1 C-type lectin-like domain. Mutation of Trp221, His223, or Tyr228 abolished this effect, indicating that the shallow hydrophobic groove formed by these residues is important for glucan recognition [106]. Molecular dynamics and docking analyses further predicted that a triple-helical β-(1→3)-glucan can form a stable endo-type complex in this groove through a hydrogen-bond network involving the C4 and C6 hydroxyl groups, whereas single-helical and double-helical configurations formed fewer stable complexes [107].

Toll-like receptors accommodate a broader range of backbone architectures and solution conformations. Fuc-Sc has a linear →3)-α-L-Fuc2S-(1→ backbone and adopts a random-coil conformation in PBS. Its stimulatory effect on macrophages involved TLR2 and TLR4, and controlled depolymerization changed the activity of the same polysaccharide [108,109]. fCS-Sc, another FCS with predominantly 2,4-disulfated fucose branches, adopts rigid rod-like chains with a single-helical structure in PBS. Fluorescent labeling showed its association with macrophages, and receptor-inhibition experiments further indicated the involvement of TLR2 and TLR4 [47,110]. Although their backbones, branches, and solution conformations are different, both polysaccharides involve the same receptor pair in macrophage recognition. Similar TLR4-associated responses were observed among six fucoidan fractions from *Saccharina japonica*. Despite differences in molecular weight and chemical composition, all six fractions increased TLR4 expression, whereas SR-A competition was observed only for two low-molecular-weight degraded fractions [111].

Overall, receptor selectivity is shaped by the combined three-dimensional presentation of the backbone, branches, charged groups, and molecular size at the cell surface. These receptor-associated differences provide an upstream basis for the subsequent activation of NF-κB and MAPK pathways. Future studies should combine detailed structural characterization with direct receptor-binding, receptor-blocking, or knockout experiments and molecular simulations to identify the features that govern receptor selectivity.

#### 3.3.2. NF-κB Signaling Pathway

**Figure 3:**
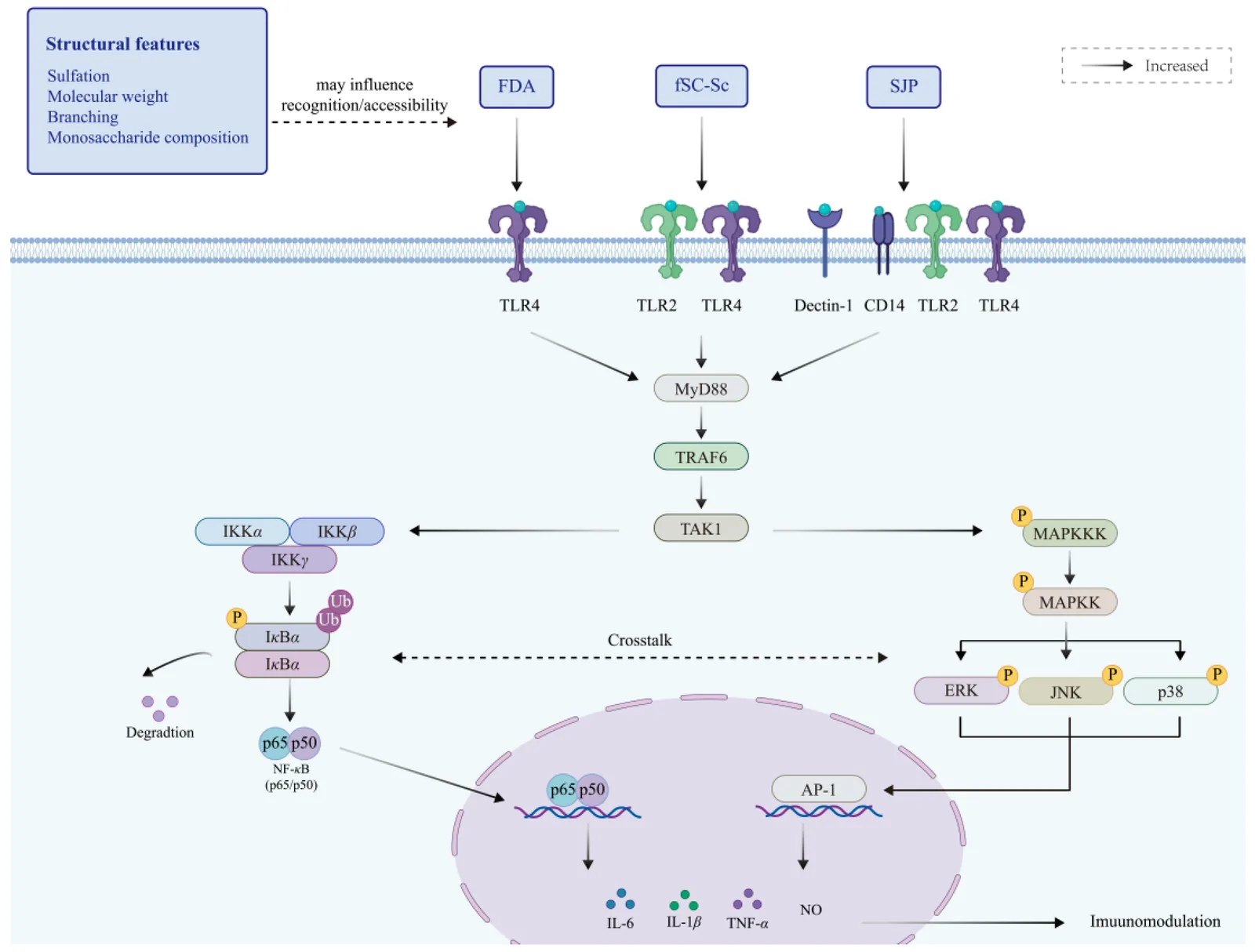
MFPs exert immunomodulatory effects through the NF-κB and MAPK pathways.

The NF-κB pathway is an important pathway involved in the immunomodulatory effects of MFPs. Activation of receptors such as TLR2 and TLR4 can initiate MyD88-dependent signaling through IRAK, TRAF6, TAK1, and the IKK complex. Subsequently, the phosphorylation and degradation of IκBα release NF-κB, allowing its translocation into the nucleus and the transcription of the immune-related genes, including those encoding IL-6, TNF-α, IL-1β, and iNOS (Figure 3).

TLR4 is frequently involved in NF-κB activation by MFPs. It can act alone or together with other receptors. FDA from *Durvillaea antarctica* induced dendritic-cell and T-cell activation in wild-type mice, whereas these responses were largely abolished in TLR4-knockout mice [97]. This directly supports a TLR4-NF-κB route through which certain MFPs regulate the dendritic cell-T-cell axis. Fucosylated chondroitin sulfate from *Stichopus chloronotus* (fCS-Sc) was recognized through both TLR2 and TLR4 and subsequently induced IκBα degradation and p65 nuclear translocation [110]. This may be attributed to their structural differences, which can broaden the receptor signals entering the same downstream pathway. This is more evident for complex heteropolysaccharides. The sulfated heteropolysaccharide SJP from *Saccharina japonica* can activate the NF-κB pathway through Dectin-1, scavenger receptors, CD11b, CD14, TLR2, and TLR4 [112]. For such a heteropolysaccharide containing several monosaccharide residues, recognition by multiple receptors may allow its different structural regions to contribute to the overall immune response. How the receptors appear to divide or share control over different components of the macrophage response remains to be further studied.

MFPs can further regulate the downstream components of the NF-κB pathway and thereby promote immune responses in different tissues. Mussel polysaccharides (MP) can significantly increase the phosphorylation of IκBα and p65 and promote NF-κB nuclear translocation, thereby reversing cyclophosphamide-induced immunosuppression [113]. Downstream immune responses activated through the NF-κB pathway also depend on the immune context. A sulfated-rhamnoglucuronan UPP from *Ulva pertusa* activated the NF-κB pathway in macrophages, promoted phagocytosis and cytokine secretion, and improved several immune-cell responses in cyclophosphamide-treated mice. In naive mice, oral UPP activated the NF-κB pathway in ileal tissues and enhanced BAFF, APRIL, TGF-β, and IgA-related responses [114,115]. This suggests that the same MFP may produce different downstream effects through the same signaling pathway in different immune contexts. Such a property may help guide the development of functional foods based on MFPs for populations with different immune needs in the future.

#### 3.3.3. MAPK Signaling Pathway

The MAPK pathway also plays an important role in the immunomodulatory effects of MFPs. Its three commonly studied branches, ERK, JNK, and p38, transmit extracellular signals and regulate immune-cell activation and cytokine production. Pattern recognition receptors, including TLR4 and Dectin-1, can initiate MAPK cascades and activate downstream transcription factors such as AP-1. By modulating these pathways, MFPs can regulate the immune responses and maintain immune homeostasis (Figure 3).

MFPs mainly regulate MAPK by altering the phosphorylation of ERK1/2, p38, and JNK1/2. The sulfated polysaccharide OPS from oyster markedly enhances the phosphorylation of all three MAPK branches and restores IL-2 production in cyclophosphamide-treated mice [116]. Similarly, SFP-αII from *Sargassum fusiforme* can also regulate the phosphorylation of multiple upstream and downstream MAPK components in all three branches of MAPK. This may be associated with its high uronic acid content [19]. These studies show that some MFPs can activate the MAPK branches simultaneously. Studies using specific inhibitors further reveal that the contributions of each branch to downstream immune responses are different.

For instance, SJP increases the phosphorylation of ERK and p38 but does not induce JNK activation. Inhibition of p38 reduced SJP-induced NO production, while inhibition of ERK mainly reduced IL-6 production [112]. For the sulfated polysaccharide PHPS from *Porphyra haitanensis*, inhibition of JNK or JAK2 reduced NO production, whereas inhibition of p38 had no significant effect [117]. These findings suggest that MFPs with different structures can selectively regulate individual MAPK branches. Even when certain MFPs activate macrophages and promote the same immune mediator, the signaling branches responsible for this response may differ.

Furthermore, extensive crosstalk exists between the MAPK and NF-κB pathways, with MAPKs serving as important upstream regulators of NF-κB. Studies on the *Strongylocentrotus nudus* egg polysaccharide SEP show that inhibition of ERK, JNK, or p38 phosphorylation significantly reduces NF-κB phosphorylation induced by SEP and suppresses the expression of cytokines [87]. This indicates that the signaling pathways regulated by MFPs do not always act independently. Their interaction can further shape the type and intensity of the immune response.

### 3.4. Structure-Immunomodulatory Activity Relationships of MFPs

The immunomodulatory effects of MFPs are jointly influenced by molecular weight, sulfation, monosaccharide composition, glycosidic linkage, branching, and higher-order organization [118]. Studies indicate that these structural factors do not act independently. Instead, they determine how polysaccharides are presented to immune cells and exhibit different immunomodulatory activities.

Sulfation is one of the most extensively studied structural factors affecting the activities of MFPs. Sulfate groups increase the negative charge density of polysaccharide chains and can strengthen electrostatic interactions with positively charged regions of proteins. In many sulfated polysaccharides, the presence of sulfate groups is important for maintaining immunomodulatory activities. A fucoidan from *Silvetia siliquosa*, which consisted mainly of sulfated fucose residues, can activate macrophages. After desulfation, its immunomodulatory activity disappeared [119]. Similarly, desulfation of KCA and KCW markedly reduced their anticomplementary activity [93]. These studies show that sulfate groups are required for the activities of these MFPs. In addition, the more highly sulfated and fucose-rich fucoidans from *Saccharina latissima* and *Laminaria hyperborea* exhibited complement inhibition, while the less sulfated, galactose-rich fucoidan from *Alaria esculenta* did not show this activity [120]. This also supports the importance of sulfation but does not separate it from fucose content and backbone composition. Therefore, it cannot be simply concluded that higher sulfate group contents correspond to stronger immunomodulatory activities of MFPs. Their activities should be evaluated in combination with other structural characteristics.

Molecular weight can affect the solubility, viscosity, diffusion and steric accessibility of MFPs. Low-molecular-weight MFPs generally show lower viscosity and better diffusion, which may facilitate their contact with cells and biological targets. Three sulfated polysaccharides from *Porphyra haitanensis* contained similar repeating units but differed in molecular weight and sulfate content. The fractions with lower molecular weights and higher sulfate contents showed greater RAW264.7 cell proliferation, phagocytosis, and NO production than the higher-molecular-weight fraction, indicating that reduced chain size combined with increased sulfation may improve macrophage activation [121]. The derivatives of fucosylated chondroitin sulfate from *Holothuria fuscogilva* showed stronger stimulating effects on macrophages than the parent high-molecular-weight polysaccharide, and molecular weight exerted a greater influence than the degrees of fucosylation and deacetylation [122]. These results suggest that moderate depolymerization can improve the immunomodulatory activity of some MFPs. However, the relationship between molecular weight and activity is not universally linear. High-molecular-weight polysaccharides from *Furcellaria lumbricalis* promoted TLR4/NF-κB-associated immune responses, while their partially depolymerized derivatives did not show the same immunomodulatory effects [123]. This may be because high-molecular-weight polysaccharides may retain stronger multivalent interactions, higher viscosity, and more pronounced local effects in the gastrointestinal tract.

Branching can influence chain flexibility, solution conformation, and the spatial presentation of functional groups. More highly branched MFPs may present a larger interaction surface or more accessible structural motifs to receptors or immune cells, which could contribute to stronger immunostimulatory activity. A homologous series of α-glucans isolated from *Hemicentrotus pulcherrimus* shared a similar α-(1→4)-glucan backbone but differed in the degree of branching. The more highly branched fractions generally induced stronger NO, TNF-α, and IL-6 production in macrophages. Among them, the HPP-6S, which is the most highly branched, showed strong immunostimulatory effects at the very low concentration of 1.95 μg/mL [81]. Fucosyl side chains are also an important structural feature of fucosylated chondroitin sulfate. FCSs containing longer or less common side chains often exhibit distinct biological properties. PP and HN, which have the rare GalNAc–Fuc disaccharide branches, display significant immunostimulatory activities. The enhanced activity may be associated with the presence of longer and more complex side chains, which could strengthen multivalent interactions with receptors on the cell surface and thereby more effectively stimulate the proliferation of lymphocytes and granulocytes [39]. The fucose branches are therefore considered one of the key structural elements influencing the biological activity of FCS. Experimental studies have shown that removal of the fucosyl branches markedly reduces or even abolishes the ability of FCS to activate macrophages and bind to selectins [124].

Monosaccharide composition also contributes to the structural and functional diversity of MFPs. Fucoidan and β-glucan isolated from the same *Laminaria japonica* produced different immune responses in mice. The fucoidan promoted splenic dendritic-cell maturation, inflammatory cytokine production, and NK- and T-cell activation, but the β-glucan laminarin showed substantially weaker effects [125]. Similarly, the sulfated polysaccharide with abundant fucose residues from *Saccharina latissima* activated murine B lymphocytes, while the glucose-rich laminaran fraction was inactive [102]. However, these comparisons do not demonstrate that fucose contributes more strongly to immunomodulatory activity than glucose, because these MFPs differed not only in their major monosaccharide compositions but also in sulfation, glycosidic linkages, molecular weight, and conformation. Current studies therefore support that fucose, sulfation, and an appropriate backbone contribute to the immunomodulatory effects together, rather than an independent effect of fucose alone. In addition, since the contribution of monosaccharide composition is more difficult to evaluate independently, methods for independently evaluating this contribution remain to be developed.

## 4. Food Applications and Challenges of MFPs

In addition to influencing biological activity, the structural characteristics of MFPs also determine their physicochemical performance in food fields. Molecular weight, charge density, chain regularity, and functional groups affect viscosity, gelation, protein interactions, film formation, and controlled release, thereby providing the structural basis for their diverse food applications.

### 4.1. Food Additives

MFPs are widely used as food additives because of their high viscosity, good gelling ability, strong water-holding capacity, and stable emulsifying properties. Because of their abundant carboxyl and sulfate groups, MFPs such as sodium alginates, carrageenan, and fucoidans can influence the rheological behavior and gel characteristics of foods by forming three-dimensional network structures of varying strengths through hydrogen bonding and ionic interactions. In meat products, dairy products, beverages, jellies, sauces, and baked goods, MFPs provide multiple functions, including thickening, stabilization, gel formation, and improvement of texture and mouthfeel.

MFPs can interact with various food components to maintain food stability and modulate structural and textural attributes. Sodium alginate and κ-carrageenan strengthen myofibrillar protein networks through electrostatic interactions and hydrogen bonding. This improves the hardness, elasticity, and water-holding capacity of meat products [126]. Sodium alginate can promote protein alignment and induce the formation of biomimetic fibrous structures during the high moisture extrusion of surimi and soy proteins, thereby enhancing the toughness and fibrous texture of meat analogues [127]. In thermally processed dairy products such as baked milk, carrageenan can also prevent protein precipitation during prolonged high temperature processing. λ-Carrageenan mainly increases viscosity and slows the sedimentation of protein aggregates, whereas κ-carrageenan and ι-carrageenan can adsorb onto the surface of whey protein casein aggregates, inhibiting further aggregation. The effect depends on carrageenan type because differences in sulfation and gel-forming ability alter its interactions with proteins [56]. This suggests that the charge density and chain organization of MFPs can be used to regulate the structure of high-protein foods.

MFPs can also protect foods against microbial growth and structural deterioration during storage. Dilysine-modified chitosan (DL-chitosan), which possesses enhanced cationic density, can exert antibacterial activity by disrupting bacterial cell membranes and exhibits broad-spectrum inhibitory effects against various microorganisms. DL-chitosan effectively inhibits bacterial growth and extends the refrigerated shelf life of chicken products [128]. Chitosan, fucoidan, and sodium alginate provide effective cryoprotective effects for frozen dough through different mechanisms. The protective effect of chitosan mainly arises from the amino and hydroxyl groups on the glucosamine units generated by deacetylation, which can form strong hydrogen bonds with water molecules and thereby inhibit ice recrystallization and crystal growth. In addition, chitosan can slow the abnormal oxidation of free sulfhydryl groups and reduce the exposure of hydrophobic regions in gluten proteins, thereby maintaining the structural integrity of the gluten network at the molecular level. Sodium alginate contains a high density of carboxyl groups with strong hydration capacity, allowing it to tightly bind free water and significantly reduce the proportion of freezable water. At the same time, physical crosslinking enhances the viscoelasticity and mechanical strength of the dough network. Fucoidan can further induce the formation of a more compact gluten protein microstructure, acting as a physical barrier that buffers the mechanical damage caused by ice crystals and thereby improving sensory quality [129,130].

**Figure 4:**
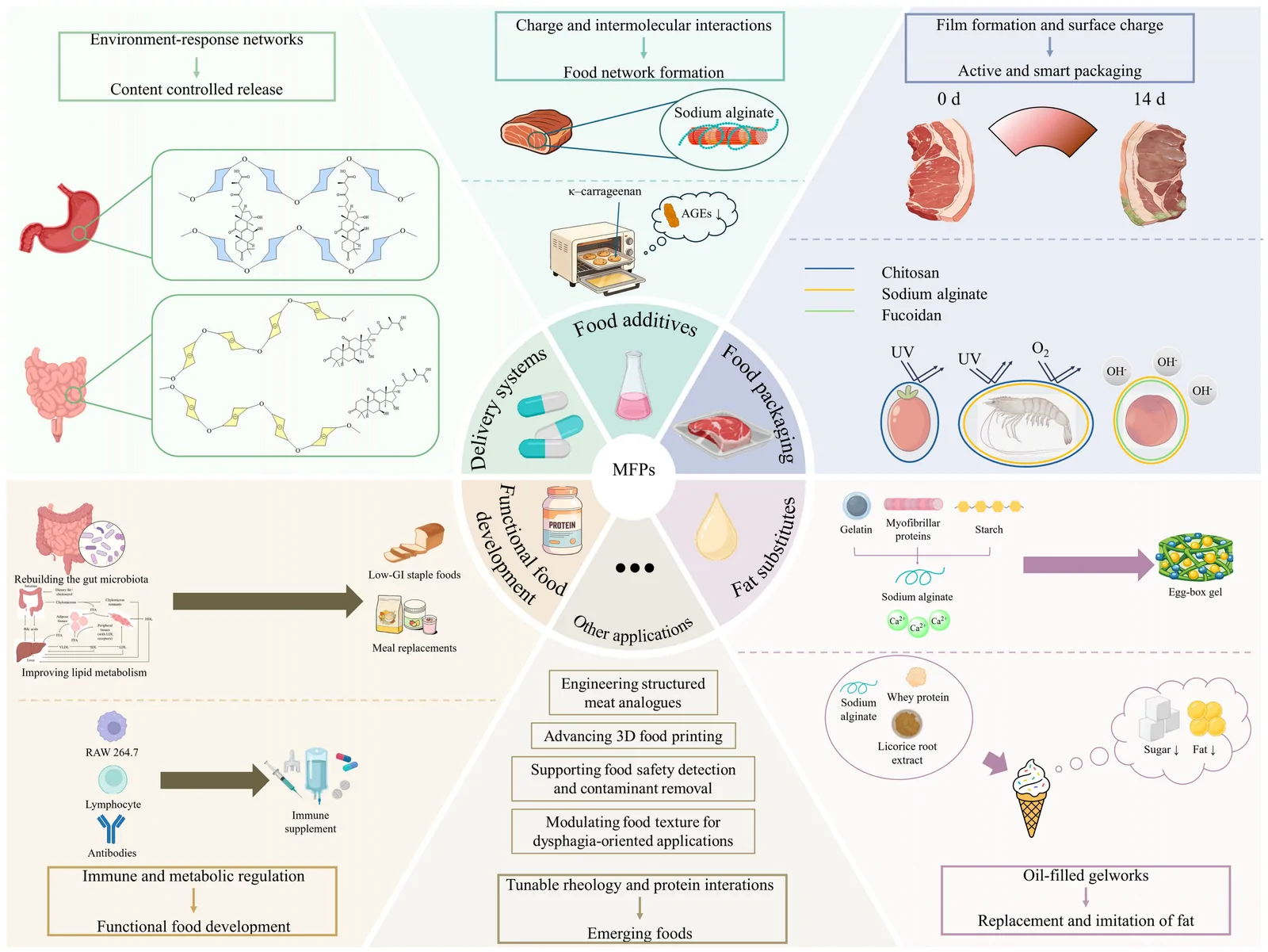
The applications of MFPs in various aspects of the food industry.

While improving food quality as additives, MFPs can also provide nutritional benefits. In heat-processed foods, κ-carrageenan has been shown to inhibit the formation of advanced glycation end-products (AGEs) by scavenging or binding key intermediates such as glyoxal (GO) and methylglyoxal (MGO). κ-Carrageenan can also improve pore structure and softness of cake, maintaining desirable sensory properties while reducing potential health risks associated with advanced glycation reactions [131]. In buckwheat extruded noodles, sodium alginate can form hydrogen-bonded networks with starch. This structure enhances thermal stability and reduces crystallinity. More importantly, the network partially limits the accessibility of digestive enzymes to starch, thereby significantly reducing in vitro starch digestibility [132]. These characteristics provide a promising strategy for developing staple foods with a lower glycemic index (GI) and improved textural quality (Figure 4).

### 4.2. Food Packaging

MFPs have attracted increasing attention in food packaging research in recent years because of their natural origin, high biosafety, and good biodegradability. Chitosan, sodium alginate, and carrageenan exhibit good film-forming properties in solution. Therefore, they have been widely investigated for use in food preservation coatings and packaging materials. MFPs provide promising alternatives to petroleum-based materials for the development of active and sustainable packaging systems [133].

Among various MFPs, chitosan is widely used in preservation films and coatings because its protonated amino groups can interact with negatively charged microbial surfaces. However, films only composed of chitosan still show limitations in mechanical strength, barrier properties, and antioxidant capacity. To overcome these drawbacks and broaden its applications, chitosan films are often modified through physicochemical approaches or combined with other materials. Studies have shown that combining chitosan with protein-polyphenol conjugates or incorporation of nanozeolite and modified lignin can improve these properties and enhance the preservation of perishable foods [134,135]. Anionic sodium alginate exhibits excellent oxygen barrier properties and can be combined with positively charged chitosan to construct multilayer packaging structures. Chitosan-sodium alginate bilayer films containing tea tree essential oil Pickering emulsions show improved barrier performance and mechanical strength, while also enabling controlled release of active compounds [136]. Fucoidan can also be used directly as an edible coating. In nectarines, fucoidan coatings reduced weight loss and delayed several storage-related quality changes [137].

Smart packaging systems based on MFPs typically employ pH-sensitive pigments, such as anthocyanins, to respond to TVB-N, ammonia, or organic acids produced during food spoilage, thereby providing real-time visual indicators of food freshness through color changes [138]. Sodium alginate has been combined with gellan gum or pullulan to immobilize anthocyanins. Hydrogen bonding within these composite networks improves film stability, while the pigments respond to pH changes during meat or seafood spoilage [138,139]. Chitosan can provide an alternative without pigment. Under specific conditions, chitosan can self-assemble into a Bouligand structure, a chiral nematic arrangement capable of producing stable structural coloration. When acidic compounds or aldehydes such as formaldehyde are generated during food deterioration, the interlayer spacing or refractive index within the chitosan structure changes, resulting in a visible shift in the structural color of the film. Because no synthetic dyes are used, this system avoids the potential risk of indicator substances migrating into food and therefore provides a safe and reliable in-package freshness indicator [140]. Moreover, chitosan is often integrated into bilayer smart indicator films to improve structural stability and indication performance, further expanding the application forms of MFPs in intelligent packaging systems [141]. Overall, in the fields of food preservation coatings and smart packaging, MFPs not only serve as environmentally friendly alternatives to petroleum-based packaging materials but also, when combined with modification strategies and bioactive components, provide important research directions for ensuring food safety and extending the shelf life of perishable foods.

### 4.3. Delivery Systems

MFPs, particularly sodium alginate, chitosan, carrageenan, and fucoidan, have gradually demonstrated significant potential in the construction of functional ingredient delivery systems due to their favorable biocompatibility, abundant reactive functional groups, and tunable physicochemical properties. MFPs can form structurally stable matrices through intermolecular interactions and can be modified via physical or chemical approaches, making them representative bio-based materials for the design of intelligent delivery systems.

Sodium alginate can form the egg-box structure through crosslinking with multivalent cations, which endows the system with pronounced responsiveness to environmental pH variations. Under acidic gastric conditions, the egg-box network remains compact. In contrast, in neutral or mildly alkaline intestinal environments, ionization of carboxyl groups induces network swelling and promotes the release of the active content [142]. Incorporating nano calcium carbonate can further reinforce the alginate egg box network, improving the survival of encapsulated probiotics during processing and gastrointestinal exposure [143]. Chitosan can also be combined with sodium alginate to form polyelectrolyte complexes through electrostatic interactions, improving mechanical stability and providing protection against oxygen and light. The complexes have been used as wall materials to microencapsulate walnut oil. This encapsulation effectively inhibits the oxidative rancidity of unsaturated fatty acids [144]. Moreover, the mucoadhesive properties of chitosan can facilitate its interaction with the intestinal mucosa and improve the absorption efficiency of bioactive contents [145].

Delivery carriers based on the unique thermoreversible gelation property of κ-carrageenan can enhance the chemical stability of heat sensitive or photosensitive compounds by restricting molecular mobility within the gel network. κ-Carrageenan beads provide thermal protection for curcumin and support its sustained, pH-dependent release under simulated gastrointestinal conditions [146]. MFP-based emulsion gel systems can be engineered to possess gastric buoyancy by regulating their density, thereby improving the bioavailability of functional ingredients with narrow absorption windows [147]. Through precise regulation of intermolecular interactions, MFP-based delivery systems can achieve coordinated protection and targeted release of multiple functional components. Overall, the ionic crosslinking, gelation, and interfacial properties of MFPs provide several routes for protecting functional ingredients and controlling their release.

### 4.4. Functional Food Development

MFPs have exhibited generally favorable safety profiles and diverse bioactivities, including effects on metabolic regulation and immune modulation. Therefore, they have shown distinctive application potential in the development of functional foods.

As discussed in Section 3, MFPs can regulate macrophages, lymphocytes, dendritic cells, and antibody production. They have shown potential in the development of immunomodulatory functional foods. A heteropolysaccharide (SZF) isolated from *Sargassum zhangii* can induce nitric oxide production in macrophages, showing potential as a raw material for immune-enhancing nutritional supplements [148]. Low-molecular-weight fucoidan (IOY) from *Ishige okamurae* significantly promotes lymphocyte proliferation and restores hematopoietic function in cyclophosphamide-induced immunosuppression models, showing promise for the development of auxiliary nutritional products or health foods for cancer patients [149]. These studies support the biological basis for immunomodulation but do not directly demonstrate benefits in healthy consumers or cancer patients. In a randomized pilot study involving healthy adults, ingestion of *Cladosiphon okamuranus* fucoidan increased NK-cell activity at eight weeks. However, the between-group effect was mainly observed in male participants, and no significant response was detected in female participants [90]. Combined with the cell and animal studies, this result indicates that the observed immune response may depend on the baseline immune state, population, dose, and type of MFP preparation. Therefore, the development of MFPs into immune regulators should define the intended population and the target immune function.

In terms of metabolic regulation, MFPs can exert multilevel effects ranging from physical modulation within the gastrointestinal tract to molecular and systemic regulation. Fucoidan can alter the gelatinization behavior of starch and reduce the accessibility of digestive enzymes. Fucoidan can also inhibit α-amylase and α-glucosidase, thereby slowing carbohydrate digestion and attenuating postprandial glycemic responses [150]. Sodium alginate can alter the hydration environment surrounding the enzyme and thereby influence its conformation and catalytic activity. As a result, sodium alginate can significantly influence the catalytic activity of enzymes and regulate disaccharide hydrolysis under specific conditions [151]. These show that MFPs can influence carbohydrate digestion within the food matrix or gastrointestinal lumen by limiting enzyme accessibility or altering enzyme activity through non-covalent interactions. Fucoidan isolated from *Sargassum fusiforme* (SFF) has been shown to modulate gut microbiota composition and suppress body weight gain and lipid accumulation in high-fat-diet-fed mice [152]. Collectively, these findings offer new strategies for the development of low-glycemic index (Low-GI) staple foods, meal replacements, and weight-management-oriented functional foods.

### 4.5. Fat Substitutes

High intake of saturated fat is associated with an increased risk of obesity and cardiovascular diseases, which has promoted the development of low-fat foods. However, maintaining desirable texture and structural stability while reducing fat content is an important problem in food formulation. Therefore, developing safe and functional fat substitutes that meet health demands without compromising texture and flavor is of great importance to the food industry.

Sodium alginate exhibits unique advantages in the construction of novel fat-mimicking systems due to its anionic nature, excellent gelling ability, and good biocompatibility. Studies have demonstrated that emulsion gels based on sodium alginate can closely mimic the key textural parameters of pork fat, including hardness, viscoelasticity, and thermal stability. In a gelatin-sodium alginate system, the two polymers formed aggregates mainly through hydrogen bonding and hydrophobic interactions, enabling the gel network to retain corn oil. The resulting emulsion gel showed stable viscoelastic properties and reduced free fatty acid release compared with free corn oil during in vitro digestion [153]. Sodium alginate can interact with other components such as gelatin, myofibrillar proteins, or starch through hydrogen bonding and hydrophobic interactions. In the presence of calcium ions, alginate chains form an egg-box network, resulting in a continuous and robust three-dimensional gel network. This network immobilizes liquid vegetable oils within the gel matrix, forming emulsion gels with semisolid textures similar to those of animal fats [154]. Both studies showed that the polysaccharide network can convert liquid oil into a stable semisolid structure and protect it during processing. However, these results were mainly obtained from model gels, and their performance in food products still needs to be verified.

Studies in actual foods show that the replacement level is closely related to the food matrix. Agar- and κ-carrageenan-based emulsion gels were used to replace pork back fat in Frankfurter sausages. Partial replacement maintained physicochemical and mechanical properties similar to those of the control, whereas complete replacement caused some changes in texture and lipid oxidation, although the products were still accepted by untrained panelists [155]. In the development of innovative low-fat dairy products, sodium alginate can form ternary stabilized foam systems when combined with whey proteins and licorice root extract. These systems can simultaneously replace fat and sugar components in ice cream formulations and limit ice crystal growth, with similar sensory properties to those of the control at a relatively low replacement level. As the replacement level increased, the ice cream became harder and its overall acceptance scores decreased [156].

Therefore, using MFPs as fat substitutes needs to balance structural stability with fat-like behavior during consumption. A strong gel network can reduce oil separation and oxidation and may delay lipid digestion. However, excessive addition of MFPs or crosslinking may produce an overly dense network, leading to increased hardness and reduced flavor release. Therefore, applications of MFPs as fat substitutes should take into account both sufficient structural stability and the sensory functions normally supplied by fat.

### 4.6. Other Applications

Beyond the applications discussed above, MFPs also show potential in emerging areas of food design and processing.

In the development of emerging meat alternatives, chitosan can synergistically interact with soy β-conglycinin amyloid fibrils through electrostatic attraction and hydrogen bonding to construct three-dimensional porous scaffolds with high mechanical strength and good thermal stability. These scaffolds provide a biomimetic microenvironment resembling the natural extracellular matrix, which supports cell adhesion and differentiation in cultivated meat systems [157]. Differences in sulfate group density among carrageenans have also been exploited to precisely regulate plant protein denaturation and alignment. ι-carrageenan, with a higher sulfate content, is more effective than κ-carrageenan in enhancing disulfide bond crosslinking and fiber anisotropy in pea protein-based meat analogues, enabling a closer replication of the texture and chewing characteristics of natural meat [158].

With advances in digital processing technologies, the application of MFPs in 3D food printing has further expanded the possibilities for personalized food design. By inducing complex coacervation between fish gelatin and fucoidan, fish oil can be efficiently encapsulated to produce fat-mimicking inks that combine thermoresponsive flow behavior with excellent antioxidant performance, thereby meeting the dual requirements of printability and nutrient delivery [159]. κ-carrageenan can still provide strong elastic support for golden thread surimi at elevated temperatures. This allows golden thread surimi to solidify through rapid microwave heating at 45–80 °C without the need for transglutaminase, significantly improving the fidelity of printed structures [160].

MFPs also show considerable potential in food texture modulation. By incorporating chitosan, or by adjusting its viscosity, steric hindrance, and electrostatic-repulsion effects, the rheological properties and microporous structure of surimi gels can be effectively regulated. This approach enables the development of soft foods that meet IDDSI levels 5–6, with improved chewability and oral processing performance, thereby enhancing eating safety and sensory experience for patients with dysphagia [161].

Overall, MFPs have shown potential in emerging food applications through their tunable molecular structures, controllable swelling and release behaviors, and favorable biocompatibility. MFPs have gradually evolved from traditional additives into key functional components, providing new approaches for personalized and sustainable food design. The wider application of MFPs will depend on whether these functions can be maintained in real food products and during large-scale processing. Their commercialization will also require careful evaluation of safety, product consistency, and regulatory requirements.

### 4.7. Safety Evaluation and Regulatory Considerations of MFPs

MFPs have shown remarkable biological activities and broad application prospects in the food industry. However, systematic safety evaluation remains an important premise for their industrialization and commercial application. As summarized in Table S1, in commonly used cell models, such as RAW264.7 macrophages, most MFPs are considered to be non-cytotoxic within the tested concentration ranges. Animal studies have also shown that oral administration of most MFPs is well tolerated, with no significant body weight loss or abnormal changes in biochemical parameters observed. However, some studies have reported that certain MFPs, such as CLP-80, may exhibit potential toxicity at high doses [103]. Therefore, future studies should establish standardized dose escalation protocols for MFPs, clarify their toxicity mechanisms, and determine key toxicological parameters, including the no-observed-adverse-effect level (NOAEL), maximum tolerated dose (MTD), and acceptable daily intake (ADI). In addition, studies on the long-term safety of MFPs are limited. Research on chronic toxicity, reproductive toxicity, and potential interactions with concomitant medications is still insufficient. Since many MFPs are intended for long-term consumption as functional food ingredients or dietary supplements, comprehensive long-term safety evaluations should be conducted before their commercial application. Future studies should integrate chronic toxicological evaluation with pharmacokinetic and metabolic studies to comprehensively clarify the metabolic fate and potential safety risks of MFPs following long-term intake.

Because MFPs are derived from marine sources, their safety is also influenced by the quality of the raw materials and the degree of purification. Although there are currently few reports of allergic reactions caused by MFPs, polysaccharide products derived from marine organisms may still present a potential risk of allergenicity if they are not sufficiently purified because of residual proteins or glycoproteins [162]. For example, polysaccharides isolated from crustaceans or mollusks may contain trace amounts of crustacean allergens, such as tropomyosin, which may trigger hypersensitivity and allergic reactions in susceptible individuals [163]. In addition, marine environments may be polluted by heavy metals and other environmental pollutants. Unprocessed seaweeds and marine invertebrates are prone to accumulating harmful elements, including inorganic arsenic (As), cadmium (Cd), lead (Pb), and mercury (Hg). During large-scale industrial extraction, these heavy metals may co-precipitate with polyanionic polysaccharides, such as fucoidan and sodium alginate, through electrostatic chelation. Therefore, industrial processing of MFPs should employ rigorously validated purification procedures and heavy metal monitoring techniques to ensure compliance with food safety regulations.

In addition to safety evaluation, product standardization and regulatory approval remain major challenges that limit the application of MFPs. The structural characteristics and biological activities of MFPs are influenced by multiple factors, including species, geographical origin, extraction methods and purification procedures. MFPs obtained from different sources or produced using different extraction processes often show significant differences in molecular weight, degree of sulfation, and monosaccharide composition. These variations complicate product standardization and increase the difficulty of regulatory evaluation. Under U.S. regulations, sodium alginate is listed among substances affirmed as GRAS under specified conditions, whereas carrageenan is permitted as a direct food additive under prescribed conditions. In the European Union, sodium alginate and carrageenan are authorized as E401 and E407, respectively [164,165,166,167]. They are widely accepted worldwide and are subject to clearly defined conditions of use. In contrast, the regulatory status of fucoidan depends on the specific proprietary extraction process and the source species. The FDA issued “no questions” letters for GRN 565 concerning a preparation from *Undaria pinnatifida* and GRN 661 concerning a concentrate from *Fucus vesiculosus*. These conclusions apply to the specified preparations and intended uses rather than to fucoidan as a whole [168,169]. However, most fucoidan products have not undergone equivalent regulatory safety evaluations. Therefore, future research should focus on developing standardized extraction and characterization methods and establishing comprehensive safety evaluation and quality control systems. More rigorous purity assessment may also be required because polysaccharides with similar molecular sizes can coexist even when conventional purity criteria are satisfied [170]. This will promote the regulatory approval and industrial application of MFPs and accelerate the translation of marine polysaccharides from laboratory research into commercial food products.

## 5. Conclusions and Perspectives

With the continuous advancement of research in food science and health-related fields, the structural diversity, complex bioactivity, and application potential of MFPs have become increasingly evident. Recent studies have revealed that MFPs derived from various marine organisms possess considerable potential in immunomodulation, metabolic regulation, and improvement of food functionality. These findings have further stimulated interest in their application in functional foods, food materials, and the health industry. However, despite the rapidly growing number of studies, the systematic relationships among structural characteristics, biological mechanisms, and functional applications of MFPs remain insufficiently understood. In particular, several key scientific questions still exist regarding advanced structural characterization, immunoregulatory networks, and the translation of functional properties into targeted applications.

At the structural level, current studies primarily focus on primary structural information, including monosaccharide composition, glycosidic linkage patterns, and the type and substitution positions of functional groups. In contrast, knowledge regarding higher-order structural features of MFPs, such as spatial conformation, dynamic behavior in solution, and potential conformational transitions under physiological conditions, remains relatively limited. In addition, existing research has largely concentrated on several MFPs with typical structures or common biological sources, whereas polysaccharides with more complex structures or rare origins have received comparatively less attention. Future investigations should therefore place greater emphasis on the elucidation of higher-order structural characteristics of MFPs. Integrating advanced analytical approaches, including two-dimensional and three-dimensional nuclear magnetic resonance spectroscopy, small-angle X-ray scattering, dynamic light scattering, cryogenic electron microscopy, molecular simulation, and computational chemistry, will provide a more comprehensive understanding of the structural basis underlying biological functions. At the same time, the development of structural modification strategies guided by functional requirements remains an important research direction. By regulating parameters such as molecular weight distribution, sulfation patterns, and the sequence arrangement of monosaccharide units, it may be possible to achieve targeted structural modification of MFPs. Strategies such as enzymatic degradation, directed sulfation, and selective desulfation can provide effective tools for fine-tuning structural features and clarifying structure-activity relationships. It must be emphasized that green and controllable degradation and modification technologies will be an important prerequisite for achieving the directional regulation and large-scale application of MFP structures.

A substantial body of evidence has demonstrated the significant immunomodulatory activity of MFPs; however, their mechanisms of action require further systematic investigation. Many current studies focus on the activation or inhibition of a single type of immune cell or an individual signaling pathway. As the immune system is a highly integrated regulatory network, the cross regulatory mechanisms of MFPs among multiple immune cell types and signaling pathways remain incompletely understood. Moreover, MFPs with different structural characteristics often exhibit bidirectional immunomodulatory effects, displaying both immune-activating and immune-suppressive activities under different conditions. The factors governing these dual regulatory effects, including dosage dependence, physiological context, and long-term safety, remain insufficiently characterized. Future research should therefore establish a more systematic framework linking “structure → receptor recognition → signaling pathways → immune phenotype”. Particular attention should be directed toward the selective recognition mechanisms between MFPs and pattern recognition receptors. It will be important to clarify the structural specificity through which receptors such as Toll-like receptors, Dectin-1, and SR-A recognize distinct polysaccharide motifs. In addition, further studies should elucidate the cross regulatory relationships among key signaling pathways, including NF-κB, MAPK, and PI3K/Akt pathways, in order to reveal the molecular basis of MFP-mediated immunomodulation. Beyond classical immune signaling pathways, increasing attention has been given to the role of gut microbiota in mediating the immunomodulatory effects of MFPs. Future studies should integrate multi-omics approaches, including metagenomics and metabolomics, together with bioinformatic analyses, to systematically investigate how MFPs influence intestinal microbial communities and their metabolic products. Such approaches will help clarify the role of gut microbiota in maintaining host immune homeostasis and provide a more comprehensive understanding of immunoregulation mediated by MFPs. Combining immune signaling studies with investigations of intestinal microecology will therefore be essential for understanding the systemic regulatory functions of MFPs.

In the food and health industries, MFPs demonstrate promising prospects both as bioactive functional ingredients and as structural components in food formulations or packaging materials. However, several technological challenges remain before their large-scale application can be fully realized. The biological functionality of MFPs in foods is highly dependent on their structural integrity and the conditions of food processing. In complex food systems, structural rearrangement or degradation of MFPs may occur as a result of thermal processing, changes in pH, or interactions with other food matrix components, which may lead to reduced bioactivity or decreased nutritional bioavailability. In addition, some MFPs, particularly highly sulfated carrageenans, remain controversial regarding their potential effects on intestinal health, which may limit their high-dose or long-term application in functional foods. Future research should focus on optimizing the stability and functional performance of MFPs through rational food structure design, the construction of composite systems, and the development of innovative processing technologies. For example, constructing polysaccharide-protein composite systems or designing targeted delivery structures may enhance the stability and bioavailability of MFPs within food matrices, thereby expanding their potential applications in functional food development. In the field of food packaging, the biodegradability and intrinsic biological activity of MFPs provide an important material basis for the development of sustainable and intelligent packaging systems. Nevertheless, further improvements in mechanical strength, barrier properties, and scalable manufacturing processes are required. Strategies such as the fabrication of nanocomposite materials, crosslinking modification, and multilayer film design may significantly improve the mechanical and gas barrier performance as well as antimicrobial stability of packaging materials based on MFPs, thereby promoting their practical application in sustainable food packaging. In addition, for certain MFPs with potential health risks, more comprehensive safety evaluation systems should be established, particularly with regard to long-term dietary exposure.

Overall, as an important class of marine-derived biomacromolecules, MFPs exhibit considerable potential in food and health-related fields. With the continuous development of marine biological resources and advances in related technologies, MFPs are expected to achieve broader applications in immunomodulation, functional foods, food materials, and the health industry. From a broader perspective, research and development of MFPs not only contribute to the high-value utilization of marine biological resources but also provide new strategies for the sustainable exploitation of marine resources. Within the framework of the big food concept, marine biological resources are increasingly recognized as an important avenue for expanding global food sources. As key functional components derived from these resources, MFPs hold unique potential for promoting nutritional health and supporting the development of the blue bioeconomy. Through interdisciplinary collaboration among natural product chemistry, food science, and nutritional science, future studies are expected to further advance research on MFPs from traditional natural product investigation toward precision nutrition and health-oriented applications.

## Figures and Tables

**Table 1:** Types, main sources, bioactivities and applications of major MFPs.

Types	Main Sources	Bioactivities	Applications in the Food Field	Ref.
Chitin	Crustacean shells; mollusks	Anticancer activity; antibacterial activity; immunomodulation; antiviral activity; tissue growth and repair	Antibacterial packaging	[9]
Chitosan	Deacetylated product of chitin	Antibacterial activity; antioxidant activity; immunomodulation; antitumor activity; anti-inflammatory activity	Food packaging films; food preservatives	[9]
Fucosylated chondroitin sulfate	Sea cucumber body wall	Anticoagulant activity; immunomodulation; antitumor activity; metabolic syndrome regulation	Functional food development	[10]
Carrageenan	Red algae such as *Kappaphycus*, *Eucheuma*, and *Chondrus*	Antitumor activity; immunomodulation; anticoagulant activity; inflammatory response	Thickener; stabilizer; fat replacer; edible films and coatings	[11]
Sodium alginate	Brown algae such as *Sargassum*	Antioxidant activity; anti-inflammatory activity; skin protection	Thickener; stabilizer; gelling agent; edible films and coatings	[12]
Fucoidan	Brown algae such as *Laminaria*	Gut microbiota regulation; metabolic disorder improvement; immunomodulation	Functional food development	[13]
Agar (mainly agarose and agaropectin)	Red algae such as *Gelidium* and *Gracilaria*	Antioxidant activity; anti-aging; skin repair	Edible films and coatings; intelligent food packaging	[14]

**Table 2:** MFPs derived from different sources and their structural characteristics.

Source	Name	Molecular Weight (kDa)	Monosaccharide Composition	Glycosidic Bond	Ref.
*Laminaria japonica*	LJP-1	272.23	Gal, GlcA, Fuc, Man, Rha, Xyl, Glc	Backbone: →4)-α-D-GlcA-(1→, →6)-β-D-Gal-(1→, →4,6)-β-D-Gal-(1→ Branches: T-β-D-Gal, →4)-β-D-Gal, →3)-α-D-Rha, T-α-D-Man, →3)-α-L-Fuc, →4)-β-D-Xyl, T-β-D-Xyl	[15]
*Laminaria hyperborea*	Fuc1	1548	Fuc, Gal	Backbone: →3)-α-L-Fuc-(1→	[16]
*Porphyra yezoensis*	F2	210	Gal, Ara, Fuc	Backbone: Alternating (1→4)-3,6-anhydro-α-L-galactopyranose and (1→3)-linked β-D-galactose or (1→4)-linked α-L-galactose 6-sulfate units	[17]
*Gracilaria lemaneiformis*	GLHP	47.6	Gal, GlcN, Fuc, Rha, Ara, Glc, Xyl, Man, Fru, Rib, GalA, GulA, GlcA, ManA	Backbone: →4)-α-D-Glc-(1→4)-α-D-Glc-(1→4)-β-D-Gal-(1→6)-α-D-Glc-(1→6)-α-D-Glc-(1→	[18]
*Sargassum fusiforme*	SFP-αII	16.5	GulA, ManA	→4)-α-GulA-(1→ and →4)-β-ManA-(1→	[19]
*Apostichopus japonicus*	SCVP-2	209.1	Fuc	→3)-α-Fuc2S-(1→3)-α-Fuc4S-(1→3)-α-Fuc-(1→	[20]
*Stichopus japonicus*	NPsj	301.75	Glc	Backbone: →4)-α-D-Glc-(1→ Branch: β-D-Glc-(1→ attached at O-6 of →4,6)-α-D-Glc-(1→ every 7–9 residues	[21]
*Hemicentrotus pulcherrimus*	HPP-1S	29,960	Glc, GlcA	Backbone: →4)-α-D-Glc-(1→ Branch: α-D-Glc-(1→6) and α-D-GlcA-(1→6) attached alternately to O-6 of backbone residues	[22]
*Crassostrea gigas*	CGPs	1080	Glc	→4)-α-D-Glc-(1→	[23]
*Sepia recurvirostra*	SIP	13.78	GlcNAc, GalNAc, GlcA, Fuc	Backbone: →4)-β-D-GlcA-(1→3)-α-L-Fuc-(1→ Branch 1: α-D-GalNAc-(1→3) on C-3 of Fuc Branch 2: α-D-GlcNAc4S6S-(1→4) on C-4 of ~20% GlcA residues	[24]
*Chlamys farreri*	CMHS	35.9	GlcA, IdoA, GlcNAc, GlcNS	Backbone: →4)-β-D-GlcA-(1→4)-α-D-GlcNAc/GlcNS-(1→ (Contains rare 3-O-sulfated GlcA)	[25]
*Mactra veneriformis*	MVPS-2	426	Glc	→4)-α-Glc-(1→4)-α-Glc-(1→4)-α-Glc-(1→2)-α-Glc-(1→4)-α-Glc-(1→4)-α-Glc-(1→4)-α-Glc-(1→	[26]
*Euphausia superba*	AKC-2	145.7	GlcNAc, GlcN	→4)-β-GlcNAc-(1→4)-β-glucosamine-(1→	[27]

## Data Availability

This study did not generate any new datasets. All data analyzed are from publicly available sources, as cited in the manuscript.

## Supplementary Materials

The following supporting information can be downloaded at https://www.mdpi.com/article/10.3390/md24080286/s1, Table S1: The immunomodulatory acting sites and mechanisms/pathways of MFPs.
