# Supplementary material for: Marine Food Polysaccharides: Representative Structural Features, Immunomodulatory Effects, and Food Applications
Source: Mar Drugs. 2026 Aug 20;24(8):286. doi: 10.3390/md24080286 (PMC13514180; doi:10.3390/md24080286)
Supplement: Supplementary file 1 [file marinedrugs-24-00286-s001.zip › marinedrugs-4475190-supplementary.pdf]

Table S1. The immunomodulatory acting sites and mechanisms/pathways of MFPs.

Acting sites	Sources	Names	Models	Doses	Main Effects	Mechanisms/Pathways	Toxicity	Ref.
Macrophage	*Caulerpa chemnitzia*	CCP	RAW264.7 macrophages	10, 30, 50, 80 μg/mL	Increases cell viability, NO production, and secretion of IL-6 and TNF-*α*	NF-κB signaling pathway and arachidonic acid metabolism pathway	No cytotoxicity	[6]
	Porphyra haitanensis	PHP	Hydrocortisone-induced immunosuppressed BALB/c mice	90 and 270 mg/kg/day	Enhanced macrophage phagocytosis and carbon clearance; increased ACP and LDH activities	TLR4/MyD88/NF-κB signaling pathway	Not reported	[80]
NK cell	*Strongylocentrotus nudus*	SEP	*In vitro*: NK-92 cells; *In vivo*: BxPC-3 and SW1990 nude mice	*In vitro*: 50-200 μg/mL; *In vivo*: 10-40 mg/kg	Enhanced NK cell cytotoxicity and activation; inhibited pancreatic cancer growth via NK cells	TLR4/MAPKs/NF-κB signaling pathway; upregulation of NKG2D/MICA	No significant toxicity; body weight unaffected *in vivo*	[87]
	Codium fragile	CFP	*In vitro*: NK cells; *In vivo*: C57BL/6 and BALB/c mice	*In vivo*: 50 mg/kg; *In vitro*: 100 μg/mL	NK cell activation; upregulation of CD69; enhanced production of IFN-γ, perforin, and granzyme B; prevented tumor infiltration	Dependent on IL-12 and DCs; potential roles of CR3 and TLR2	No significant body weight changes	[86]

Acting sites	Sources	Names	Models	Doses	Main Effects	Mechanisms/Pathways	Toxicity	Ref.
Complement system	*Kjellmaniella crassifolia*	KCA, KCW	1:10-diluted normal human serum	KCA/KCW plateaued at 10 μg/mL in the classical-pathway assay; AP50: 4.83 μg/mL (KCA) and 18.60 μg/mL (KCW)	Inhibited classical- and alternative-pathway-mediated hemolysis	Sulfate-dependent anti-complementary activity; lower molecular weight weakened the activity	Not reported	[93]
	Chondrus armatus	λ/μ/ν-carrageenan	Normal human serum	0.002-2 mg/mL	Reduced C4 deposition on mannan-coated wells by approximately 30%	Sulfation-dependent reduction of C4 deposition; possible interference with MBL and/or MASP-2	Not reported	[94]
T cell	*Undaria pinnatifida*	Low Molecular Weight Fucoidan (LMWF)	*In vitro*: Mouse bone marrow-derived dendritic cells (BMDCs)<br>*In vivo*: ICR mice	*In vitro*: 20-100 μg/mL<br>*In vivo*: 20-80 mg/kg	Promotes allogeneic $CD4^+$ and $CD8^+$ T cell proliferation; enhances IFN-γ production	Activates TLR4-mediated MAPK (ERK, JNK, p38) and NF-κB pathways in DCs	No toxicity observed in liver/kidney function tests	[96]

Acting sites	Sources	Names	Models	Doses	Main Effects	Mechanisms/Pathways	Toxicity	Ref.
	Saccharina japonica	SJP	*In vitro*: RAW 264.7 macrophages<br>*In vivo*: BALB/c mice (CTX-induced immunosuppression)	*In vitro*: 0.06-100 μg/mL<br>*In vivo*: 50-200 mg/kg	Improves CD4+ and CD8+ T cell populations in spleen<br>Increases serum IL-6 and IL-12 levels	Activates macrophages via Dectin-1, SR, CD14 receptors and MAPK/NF-κB pathways	No adverse effects on body weight or organ indices	[112]
B cell	*Ulva conglobata*	UCP	BALB/c mice (immunosuppressed by cyclophosphamide)	15, 30, 60 mg/kg	Increased the levels of serum immunoglobulins IgG, IgE, and IgM in a dose-dependent manner	Not reported	No reported toxicity	[100]
	Caulerpa lentillifera	CLP-80	BALB/c mice	150 mg/kg/day for 21 days	Increased serum IgA and IgG levels	Promotion of B-lymphocyte activity	The high dose showed potential toxicity	[103]
